# BCG vaccination mitigates tau pathology and restores cognitive function in PS19 mice

**DOI:** 10.1126/sciadv.aej4603

**Published:** 2026-10-21

**Authors:** Somnath Shee, Meixiang Huang, Meghraj Singh Baghel, Yuxin Zheng, Shichun Lun, Santosh K. Yadav, Nirbhay N. Yadav, Carlos E. Ruiz-Gonzalez, Sandeep Tyagi, Eric Nuermberger, Sanjay K. Jain, Zaver M. Bhujwalla, Barbara S. Slusher, Philip C. Wong, William Bishai

**Affiliations:** ^1^ Center for Tuberculosis Research, Division of Infectious Diseases, School of Medicine, Johns Hopkins University, Baltimore, MD, USA.; ^2^ Johns Hopkins Drug Discovery, Johns Hopkins University School of Medicine, Baltimore, MD, USA.; ^3^ Department of Neurology, Johns Hopkins University School of Medicine, Baltimore, MD, USA.; ^4^ Department of Pathology, The Johns Hopkins University School of Medicine, Baltimore, MD, USA.; ^5^ Russell H. Morgan Department of Radiology and Radiological Science, Johns Hopkins University, Baltimore, MD, USA.; ^6^ F.M. Kirby Research Center for Functional Brain Imaging, Kennedy Krieger Institute, Baltimore, MD, USA.; ^7^ Department of Oncology, Sidney Kimmel Comprehensive Cancer Center, Baltimore, MD, USA.; ^8^ Department of Radiation Oncology and Molecular Radiation Sciences, Johns Hopkins University School of Medicine, Baltimore, MD, USA.; ^9^ Psychiatry and Behavioral Sciences, Johns Hopkins University School of Medicine, Baltimore, MD, USA.; ^10^ Department of Physiology, Pharmacology and Therapeutics, Johns Hopkins University School of Medicine, Baltimore, MD, USA.; ^11^ Department of Oncology, Johns Hopkins University School of Medicine, Baltimore, MD, USA.; ^12^ Department of Neuroscience, The Johns Hopkins University School of Medicine, Baltimore, MD, USA.

## Abstract

Retrospective studies in patients with nonmuscle invasive bladder cancer have reported a significant reduction in Alzheimer’s disease incidence (12 to 78%) among Bacille Calmette-Guérin (BCG) recipients versus controls. To investigate the underlying mechanisms, we evaluated BCG in the PS19 mouse model of tauopathy. We found that BCG administration reduced hippocampal phospho-tau and microgliosis while preserving neuronal markers. In vivo volumetric T2–magnetic resonance Imaging (MRI) demonstrated attenuation of brain atrophy accompanied by increased glutamate-weighted chemical exchange saturation transfer–MRI signals. Functionally, BCG-treated mice showed improved performance in the novel object recognition test, as well as improved body-weight maintenance and survival. Transcriptomic profiling of the hippocampus revealed near complete normalization of the PS19 disease-associated gene expression signature toward that of healthy controls. Flow cytometric profiling of brain myeloid populations demonstrated a reduction in activated resident microglia, but total microglia cells remain elevated. Moreover, an increase of trained immunity marker CD80 on the recruited peripheral myeloid cells ensues following BCG treatment. Consistent with this shift in myeloid state, primary brain myeloid cells from BCG-treated mice also exhibited enhanced phagocytosis of fluorescein isothiocyanate-labeled tau fibrils and increased lactate production. Together, these findings indicate that BCG induces systemic and central nervous system myeloid cell reprogramming that limits neuroinflammation, enhances tau clearance, and rescues cognitive and neurodegenerative phenotypes in a tauopathy model. BCG is a safe, readily available therapy that merits consideration as a preventive agent against dementia.

## INTRODUCTION

There is a growing appreciation that vaccines may have beneficial off-target effects such as reducing the risk of dementia. Recent, well-publicized studies of shingles vaccines have revealed compelling evidence that immunization against varicella-zoster virus may reduce the incidence of dementia. Capitalizing on age-based eligibility for zoster vaccination in Wales, it was shown that receipt of the live-attenuated zoster vaccine was associated with a 20% reduction in incident dementia over 7 years (*1*). Subsequent studies confirmed this same observation with the recombinant zoster vaccine (*2*, *3*). In addition, it has also been shown that shingles vaccination may influence multiple stages of the dementia disease course (*4*).

While less publicized, there is also considerable evidence that Bacille Calmette-Guérin or BCG, the live attenuated vaccine to prevent tuberculosis (TB) and treat bladder cancer, may also have dementia-preventive properties. To date, six retrospective studies among patients with nonmuscle invasive bladder cancer (NMIBC) have each found a statistically significant reduction in the incidence of dementia in BCG recipients compared with controls who did not receive BCG (table S1) (*5*–*10*). Across these six studies, the reductions ranged from 12 to 78%, and the total number of patients monitored was >85,000 with mean follow-up times of 3 to 15 years. Three meta-analyses confirmed the significance of the retrospective bladder cancer studies (*11*–*13*). In addition, a 49-patient prospective trial of cognitively unimpaired older adults found that two intradermal doses of BCG over 4 weeks improved an objective blood biomarker measured 9 months later (*14*).

BCG and other vaccines have been shown to stimulate protection against heterologous antigens by a process known as trained immunity. Upon exposure to a first antigen such as BCG, innate immune cells, typically myeloid cells, have been shown to undergo epigenetic and metabolomic reprogramming such that upon exposure to a second, unrelated antigen, their immune response is greater than it was to the first (*15*–*17*). Trained immunity has been postulated as a mechanism by which BCG may protect against dementia. In this regard, it is noteworthy that a recent study of chemically induced demyelination in aged C57BL/6 mice found that intravenous BCG administered 1 month before the injury markedly accelerated healing via a mechanism involving H3K27ac and H3K4me3 epigenetic activation marks in the microglial/peripheral myeloid cells (*18*).

To directly test BCG as a preventative therapy for Alzheimer’s disease (AD), we used the PS19 (P301S) mouse model of tauopathy (*19*–*21*). This transgenic mouse expresses the P301S mutant form of the human microtubule-associated protein tau (MAPT; 1N4R) on chromosome 3 under the control of the mouse *Prnp* promoter (*19*). The overexpression of P301S tau is associated with AD-relevant pathological and functional features, including neuronal loss, microgliosis, neurofibrillary tangles (NFT) deposition, and cognitive impairment (*20*, *21*). Moreover, aberrant microglial activation is both a driver and consequence of tau pathology in this model. As phospho-tau (p-tau) accumulates in the PS19 mouse brains, microglial cells are seen to transition from a homeostatic, surveillant state to an activated phenotype that amplifies proinflammatory cytokine signaling, complement cascades, and contributes to synaptic loss (*19*, *22*–*25*). Because microglia and peripheral monocytes are central to NFT clearance and maintenance of neuronal homeostasis (*26*, *27*), we hypothesized that BCG-mediated training of myeloid cells may rebalance their state from the pathologic chronically activated state to an efficient phagocytic and homeostatic state and that such transitions may delay neuronal loss in the model.

In this study, we show that subcutaneous BCG administration mitigates hallmark features of tauopathy in PS19 mice. BCG reduced hippocampal p-tau burden and microgliosis while preserving neuronal markers. BCG-treated PS19 mice preserved hippocampal and ventricular volume, as well as displayed improved survival, body weight, and recognition-memory performance. This protective effect correlated with a BCG-driven myeloid cell reprogramming toward enhanced glycolysis and phagocytic capacity, higher costimulatory CD80 expression on peripheral myeloid cells with concomitant dampening of microglial activation and proinflammatory cytokine signaling. In sum, our data show that BCG significantly reduces tau pathology in the PS19 model and provides a mechanistic basis for BCG-mediated AD mitigation.

## RESULTS

### Subcutaneous BCG administration reduces hippocampal p-tau deposition and microgliosis, while preserving neuronal integrity

The B6;C3-Tg(Prnp-MAPT*P301S)PS19Vle/J or PS19 mouse model has been reported to display p-tau seeding at 3 months of age, neuronal loss and microglial activation at 6 months of age, and prominent NFT deposition at 6 to 9 months of age (*19*). The median survival time of PS19 mice is 10 to 12 months, indicating aggressive tauopathy (*19*, *28*). To examine whether BCG therapy may attenuate disease progression in this model, we injected hemizygous PS19 mice either with phosphate-buffered saline (1× PBS; sham) or with BCG [2 × 10^6^ colony-forming units (CFU)] subcutaneously in the dorsal neck (scruff) region. Age-matched, non-PS19 littermate mice (noncarriers) were sham treated and represent untreated, age-matched healthy controls (AMC UT). Three monthly BCG doses were administered starting at 3 months of age, followed by a no-treatment phase of 3 to 4 months (Fig. 1A). Previous studies have demonstrated that BCG-induced trained immunity correlates cytokine response and protective effects diminish progressively over time in both humans (in months to years) and mice models (weeks to months duration) (*29*–*32*). Therefore, we reasoned that a repeated vaccination protocol given at 4-week intervals will maintain sustained immune activation throughout the experimental period. The hippocampus and prefrontal cortex are the major regions affected by the tauopathy in the PS19 model (*19*). Therefore, mice were euthanized at 9 to 10 months of age, and disease in the hippocampus and cortex was assessed by immunohistochemistry (IHC) with antibodies directed against pSer202/pThr205 p-tau (AT8 anti–p-tau antibody), Iba1 (microglial activation marker), glial fibrillary acidic protein (GFAP) (astrocyte activation marker), and NeuN (neuronal integrity marker) (fig. S1 and Fig. 1). While p-tau depositions in the hippocampus were higher in hemizygous PS19 mice relative to age-matched controls (48-fold increase; *P* < 0.0001), BCG treatment significantly reduced the percent AT8 stained area (5-fold reduction relative to that of sham-treated PS19 animals; *P* = 0.0002) (Fig. 1B). In addition, BCG treatment also significantly reduced microglial activation (an indicator of neuroinflammation) as measured by the percent Iba1^+^ stained area in the hippocampus CA1 region (four-fold reduction relative to that of sham-treated PS19 mice; *P* = 0.002), and restored homeostatic state of microglia (Fig. 1C and fig. S2A). BCG also served to prevent neuronal death as demonstrated by the increased percent NeuN^+^ stained area in the hippocampal CA1 region and cortex, compared with sham-treated PS19 mice (Fig. 1E and fig. S2, B and C). BCG therapy did not reduce astrocyte activation as measured by GFAP immunoreactivity that also occurs in the hippocampus of PS19 mice (fig. S2, D and E). Taken together, the IHC data show that BCG administration ameliorated tauopathy disease progression, neuroinflammation, and associated neuronal death in PS19 mice.

**Fig. 1.**
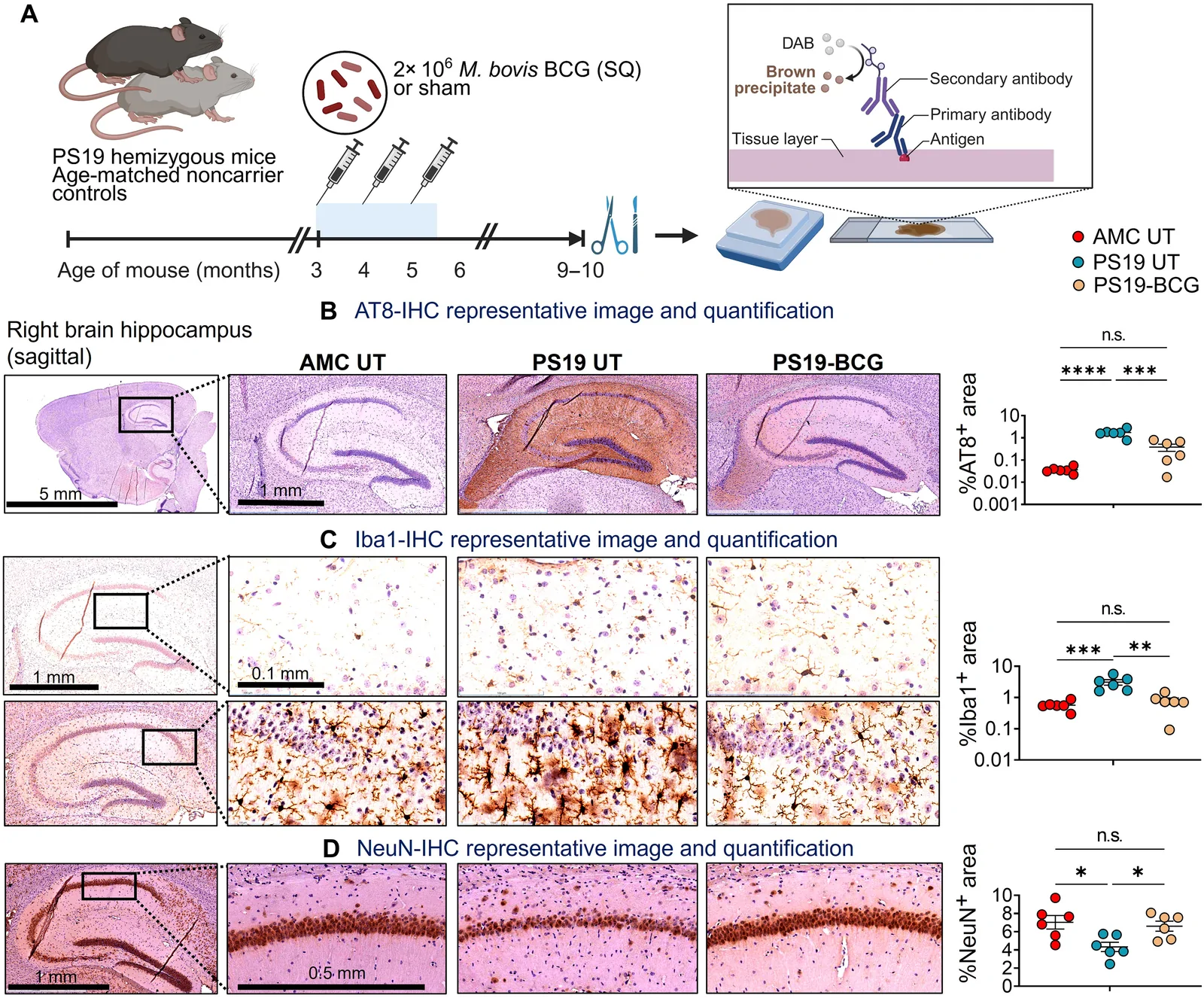
*M. bovis* BCG treatment ameliorates p-tau accumulation, microgliosis, and neurodegeneration in PS19 mice. (**A**) Schematic of the experiment (SQ, subcutaneous injection). Created in BioRender. Shee, S. (2026) https://BioRender.com/kanmk73. (**B**) %AT8^+^ area in the hippocampus of age-matched control [AMC untreated (UT)], Sham (PBS), or BCG-treated PS19 mice, as determined by ImageJ analysis (*n* = 6 per group). (**C**) %Iba1^+^ area in the hippocampus-CA1 region of AMC UT, Sham (PBS), or BCG-treated PS19 mice, as determined by ImageJ analysis (*n* = 6 per group). (**D**) %NeuN^+^ area in the hippocampus-CA1 region of AMC UT, Sham (PBS), or BCG-treated PS19 mice, as determined by ImageJ analysis (*n* = 6 per group). The data are means ± SEM and representative of two independent experiments. For each mouse brain sample, IHC was done in three planes (10-μm-thick sections) for all the antibodies. Each data point represents a mouse. Statistical analysis was calculated by one-way analysis of variance (ANOVA) with Tukey’s multiple comparisons test (n.s., *P* > 0.05; *P < 0.05; **P < 0.01; ***P < 0.001; ****P < 0.0001).

### BCG treatment prevents weight loss and extends survival of PS19 mice

Microglial activation and neuronal cell loss are closely associated with morbidity such as reduced mobility and weight loss, and these lead to early mortality in the PS19 model (*19*). By 8 to 9 months of age, the body weights of hemizygous PS19 mice show significant reductions relative to the age-matched noncarrier controls, and the difference increases with age (*28*). Moreover, the PS19-tau pathology is more severe in males than females, and hence, males lose weight faster and have shorter survival times than females (*28*). We therefore tested whether BCG treatment influences body weight and survival in two independent mouse cohorts (Fig. 2A). In cohort 1 (25 male and 30 female PS19 mice), we administered PBS (sham) or 2 × 10^6^ CFU of BCG to hemizygous PS19 mice in three monthly doses. As expected, sham-treated PS19 mice displayed reduced survival (*P* = 0.06) and failed to maintain body weight compared with age-matched controls through 9 months of age (Fig. 2, B and C). In contrast, BCG treatment reversed the accelerated mortality phenotype (*P* = n.s. compared to age-matched controls; Fig. 2B) and led to a gain of body weight at 5 to 6 (8%) and 7 to 8 (15%; *P* = 0.02) months of age relative to the sham-treated PS19 mice (Fig. 2C).

**Fig. 2.**
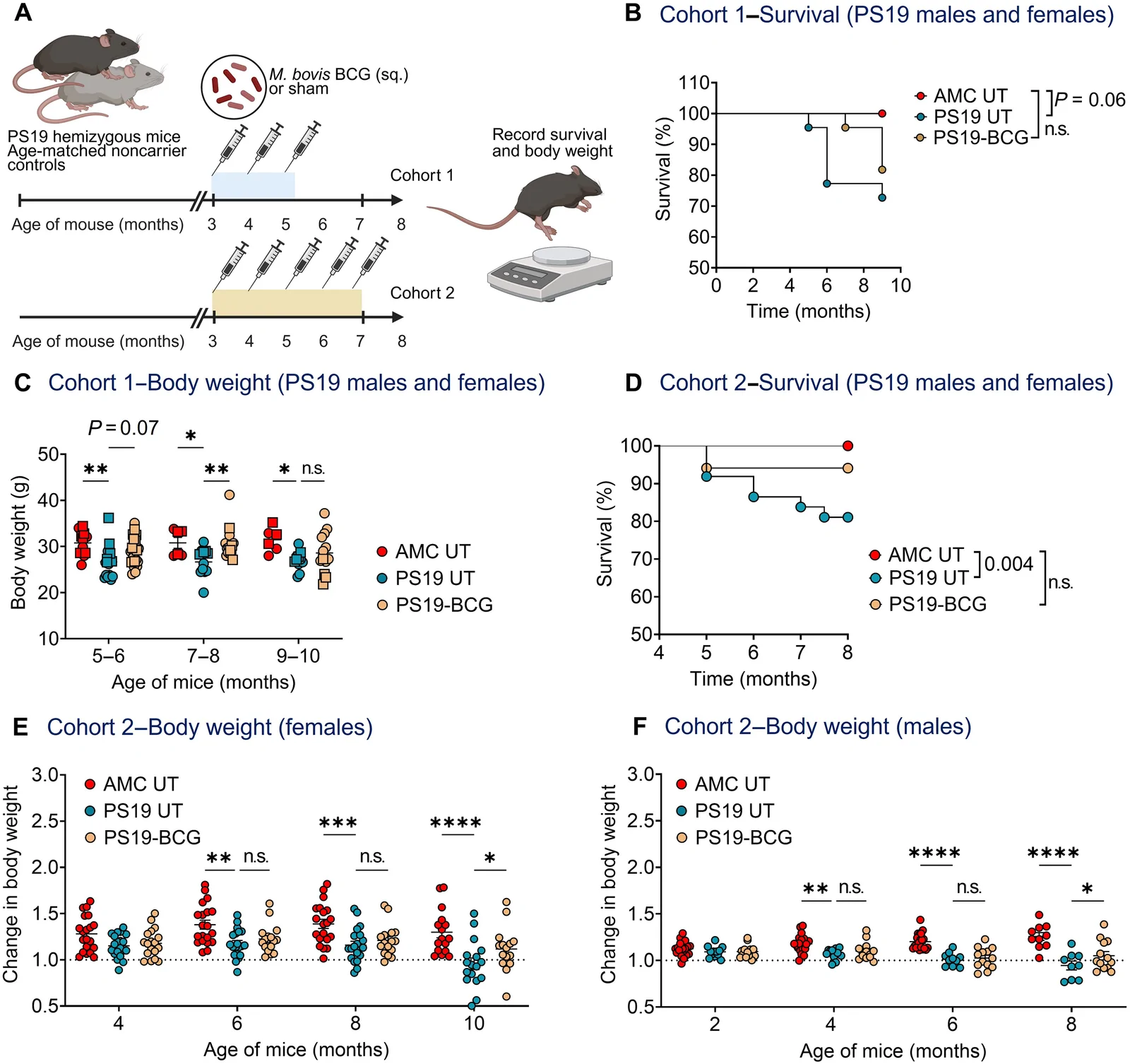
*M. bovis* BCG treatment is safe and improves body weight and survival of PS19 mice. (**A**) Schematic of the experiment. Created in BioRender. Shee, S. (2026) https://BioRender.com/kanmk73. Cohort 1 data: (**B**) Kaplan-Meier survival curve of age-matched controls (*n* = 11), and sham-treated (*n* = 22) and BCG-treated (*n* = 22) PS19 male and female mice. Indicated *P* values are measured by log-rank (Mantel-Cox) test. (**C**) Changes in body weight of the surviving mice. In cohort 2, PS19 mice were treated with five monthly doses of BCG. (**D**) Kaplan-Meier survival curve of age-matched controls (*n* = 40) and sham-treated (*n* = 37) and BCG-treated (*n* = 34) PS19 male and female mice. Indicated *P* values are measured by log-rank (Mantel-Cox) test. Changes in body weight of the surviving (**E**) females and (**F**) males from cohort 2. The data are means ± SEM. Each data point represents a mouse. Statistical analysis was calculated by two-way ANOVA with Dunnett’s multiple comparisons test (n.s., *P* > 0.05; *P < 0.05; **P < 0.01; ***P < 0.001; ****P < 0.0001).

Since BCG injections are safe both in newborn humans and mice (*17*), we increased the dosage of BCG in the cohort 2 (52 male and 59 female PS19 mice) to determine whether treatment outcomes are dose dependent. We administered PBS (sham) or 10 × 10^6^ CFU of BCG to hemizygous PS19 mice in five monthly doses. Similar to cohort 1, BCG significantly improved survival of PS19 mice (6% died; *P* = ns relative to AMC UT) (Fig. 2D). Moreover, BCG-treated PS19 mice displayed body weight gain (10 to 20%) relative to the sham-treated PS19 mice at 10 (Fig. 2E; females) and 8 months of age (Fig. 2F; males). In sum, the magnitude of body-weight improvement was broadly similar across the two treatment regimens.

### BCG treatment prevents hippocampal atrophy and reduces ventricular dilatation

Tauopathy in PS19 mice follows a close trajectory to tauopathy syndromes seen in humans with the mice developing significant volume loss of the hippocampus, amygdala, hypothalamus, and cortex, with associated ventricular dilatation by 9 months of age (*19*, *33*, *34*). We examined whether BCG administration may prevent brain atrophy in PS19 mice using T2-weighted magnetic resonance imaging (T2-W-MRI). We focused on hippocampal atrophy and ventricular dilatation and used the animals in cohort 1 and cohort 2 (Fig. 2A) for these live-imaging studies. T2-W-MRI with 6- to 7-month-old PS19 mice from cohort 1 or age-matched controls did not reveal significant differences in the hippocampus volume (fig. S3, A and B); although we observed a modest increase in ventricular volume in sham-treated 6- to 7-month-old PS19 mice that was reduced by BCG therapy, these changes were not statistically significant (fig. S3B). At this time point, we observed reduced total human tau (h-tau) protein levels (*35*) in serum from the BCG-treated PS19 mice (fig. S3C; *P* = 0.008). In cohort 2, we increased the BCG dose by subcutaneously administering PBS (sham) or BCG (10 × 10^6^ CFU) into hemizygous PS19 mice in five monthly doses. As expected, we observed distinct ventricular dilatation in 9-month-old PS19 females, as shown as high-intensity areas in the T2-W axial sections (Fig. 3, A to D). In addition, hippocampal atrophy was evident both in the axial sections and in three-dimensional (3D) reconstructions in sham-treated PS19 females (Fig. 3, C and D). Volumetric analysis by ITK-SNAP software (*36*) indicated a 21% loss of hippocampal volume and a 63% increase in ventricular volume in sham-treated PS19 females, both of which were reversed by BCG treatment (Fig. 3B). Because of the early onset of paresis in sham-treated PS19 males, we evaluated atrophy in males at 8 months of age (fig. S3, D to F). While we did not see changes in hippocampus volume at this time point, we see ventricular dilatation in untreated PS19 males that was significantly reduced in PS19 males treated with BCG (fig. S3F).

**Fig. 3.**
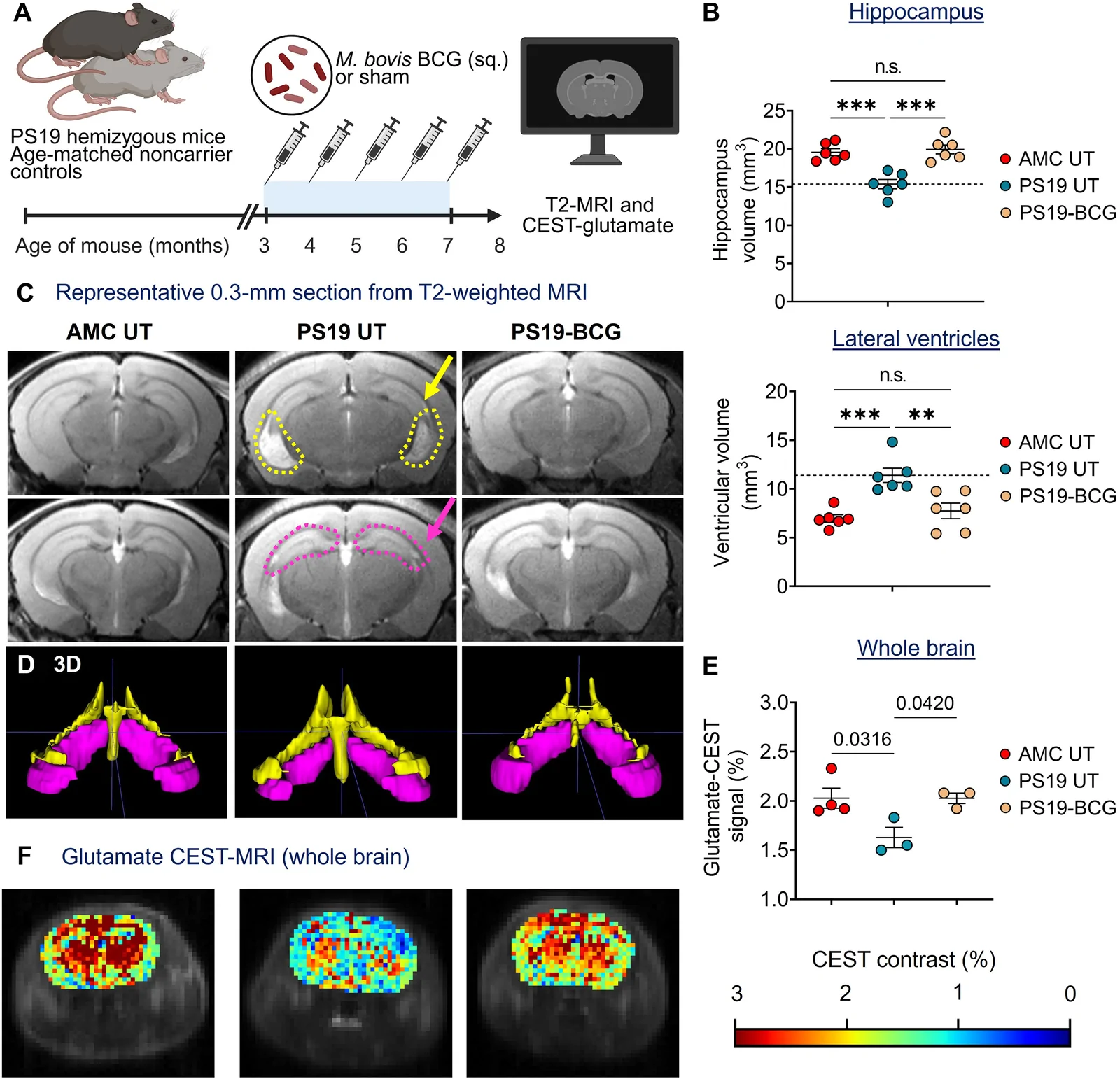
*M. bovis* BCG treatment prevents atrophy in the brain of PS19 mice. (**A**) Schematic of the experiment. Created in BioRender. Shee, S. (2026) https://BioRender.com/kanmk73. (**B**) Quantitative analysis of the hippocampus and lateral ventricle volume as determined by using ITK-SNAP software (*n* = 6 per group). Statistical analysis was calculated by one-way ANOVA with Tukey’s multiple comparisons test. (**C**) Representative 0.3-mm section of in vivo T2-W-MRI illustrating significant atrophy of hippocampus (encircled with magenta) and ventricular dilatation (encircled with yellow) in 8- to 9-month-old PS19 mice. Yellow and pink arrows indicate ventricular enlargement and hippocampus atrophy, respectively, in sham-treated PS19 mice. (**D**) Representative 3D rendering of hippocampus and lateral ventricles, as done by ITK-SNAP software. (**E** and **F**) Representative image of glutamate-weighted chemical exchange saturation transfer (CEST-MRI) maps of the brain (*n* = 3 mice per group) and quantification. The data are means ± SEM and representative of two independent biological experiments. Each data point represents a mouse. Statistical analysis was calculated by one-way ANOVA with Dunnett’s multiple comparisons test (n.s., *P* > 0.05; **P < 0.01; ***P < 0.001).

Recent studies have demonstrated that glutamate levels decrease as early as 7 months of age in live PS19 mice, and this change can be measured in vivo by an MRI method called glutamate-weighted chemical exchange saturation transfer (GluCEST-MRI) imaging (*37*, *38*). This noninvasive imaging method revealed significant 15 to 30% decreases in glutamate levels in the hippocampus and thalamus regions in PS19 mice (*37*, *38*). Therefore, we measured glutamate levels in randomly selected mice from each treatment group by GluCEST-MRI. We found that the whole brain mean GluCEST signal was significantly lower in the sham-treated PS19 mice relative to age-matched controls and that BCG administration restored the GluCEST signal to that seen in healthy, untreated non-PS19 mice [both cohort 1 (fig. S3, G and H) and cohort 2 (Fig. 3, E and F)]. Together, these noninvasive imaging data show that BCG treatment prevents tauopathy-driven brain atrophy and allows maintenance of normal glutamate levels in the PS19 mice.

### Subcutaneous administration of BCG alleviates cognitive defects

Given that BCG treatment reduces pathological tau burden, preserves neuronal integrity (NeuN), and dampens microglial activation (Iba1) and that these changes are accompanied by reduced brain atrophy by MRI, we next assessed whether these neurological benefits translate into improvements in cognitive performance. To evaluate the functional impact of BCG treatment on recognition memory, we performed behavioral testing using the novel object recognition test (NORT) (*33*, *39*). PS19 hemizygous mice and age-matched noncarrier (AMC) controls were assessed at 8 months of age, following 5 months of vehicle or BCG treatment (Fig. 4A). Locomotor activity was first evaluated using the open-field test (OFT). Total beam breaks over 30 min did not differ significantly among groups (Fig. 4B), indicating preserved motor function and comparable exploratory activity across cohorts. Recognition memory was then assessed using the NORT. Untreated PS19 mice showed a significant reduction in recognition index compared with AMC controls (*P* = 0.007), consistent with impaired novel object recognition memory (Fig. 4C). BCG treatment significantly improved recognition performance of PS19 mice, restoring behaviors to levels approaching those of healthy AMC controls (*P* < 0.05; Fig. 4C). Similarly, the discrimination index was reduced in untreated PS19 mice compared with AMC controls (*P* = 0.006) and was significantly rescued by BCG treatment (*P* < 0.05; Fig. 4D). Together, these data demonstrate that BCG-mediated immunomodulation confers robust behavioral and cognitive improvements in a tauopathy mouse model without affecting locomotor function.

**Fig. 4.**
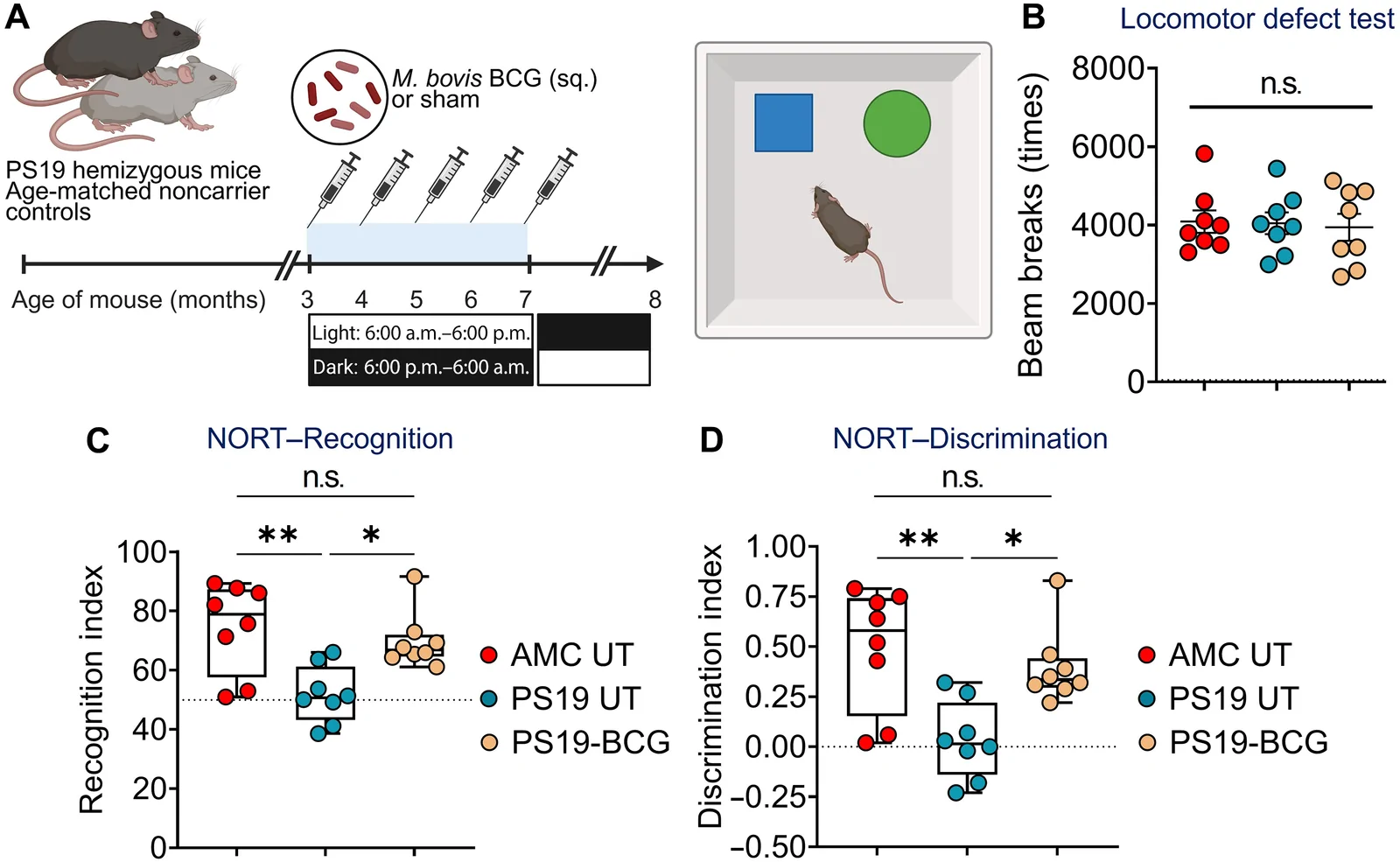
Subcutaneous *M. bovis* BCG treatment improves cognitive function in PS19 mice. (**A**) Schematic of the experiment. Created in BioRender. Shee, S. (2026) https://BioRender.com/kanmk73. (**B**) Total beam breaks in the OFT. (**C**) Recognition index and (**D**) discrimination index in the NORT. The data are means ± SEM and representative of two independent experiments. Each data point represents a mouse. Statistical analysis was calculated by one-way ANOVA with Dunnett’s multiple comparisons test (B) and Kruskal-Wallis test with Dunn’s multiple comparison test [(C) and (D)] (n.s., *P* > 0.05; *P < 0.05; **P < 0.01).

### BCG treatment restores a homeostatic transcriptional landscape and reduces proinflammatory signaling

To decipher cellular mechanisms that may explain the BCG-mediated protective effects, we investigated the transcriptomics of hippocampal tissue in our studies; we selected the hippocampus because it is the region most heavily affected by the tauopathy process in PS19 mice (*19*, *34*). Sham- and BCG-treated PS19 mice as well as untreated, age-matched, noncarrier controls who received monthly treatments at 3, 4, and 5 months of age were euthanized at age ∼9.5 months (Fig. 5A). Excised hippocampus tissue was used to isolate total RNA, and bulk RNA sequencing was performed. As may be seen in Fig. 5B, untreated PS19 mice (three middle columns) showed a markedly different transcriptional pattern than untreated, healthy, non-PS19 AMC mice (three left columns). Subcutaneous BCG treatment of PS19 mice 4.5 months prior fully restored the transcriptional pattern (three right columns) to essentially the same as that seen in untreated, healthy, non-PS19 AMC mice (three left columns). Indeed, the two transcriptomes were so similar that in comparing BCG-treated PS19 mice to the AMC mice, there were only nine differentially expressed genes (DEGs; *P*.adj. < 0.05, log_2_fold change >1 or <−1): three up-regulated genes and six down-regulated genes (Supplementary data S1).

**Fig. 5.**
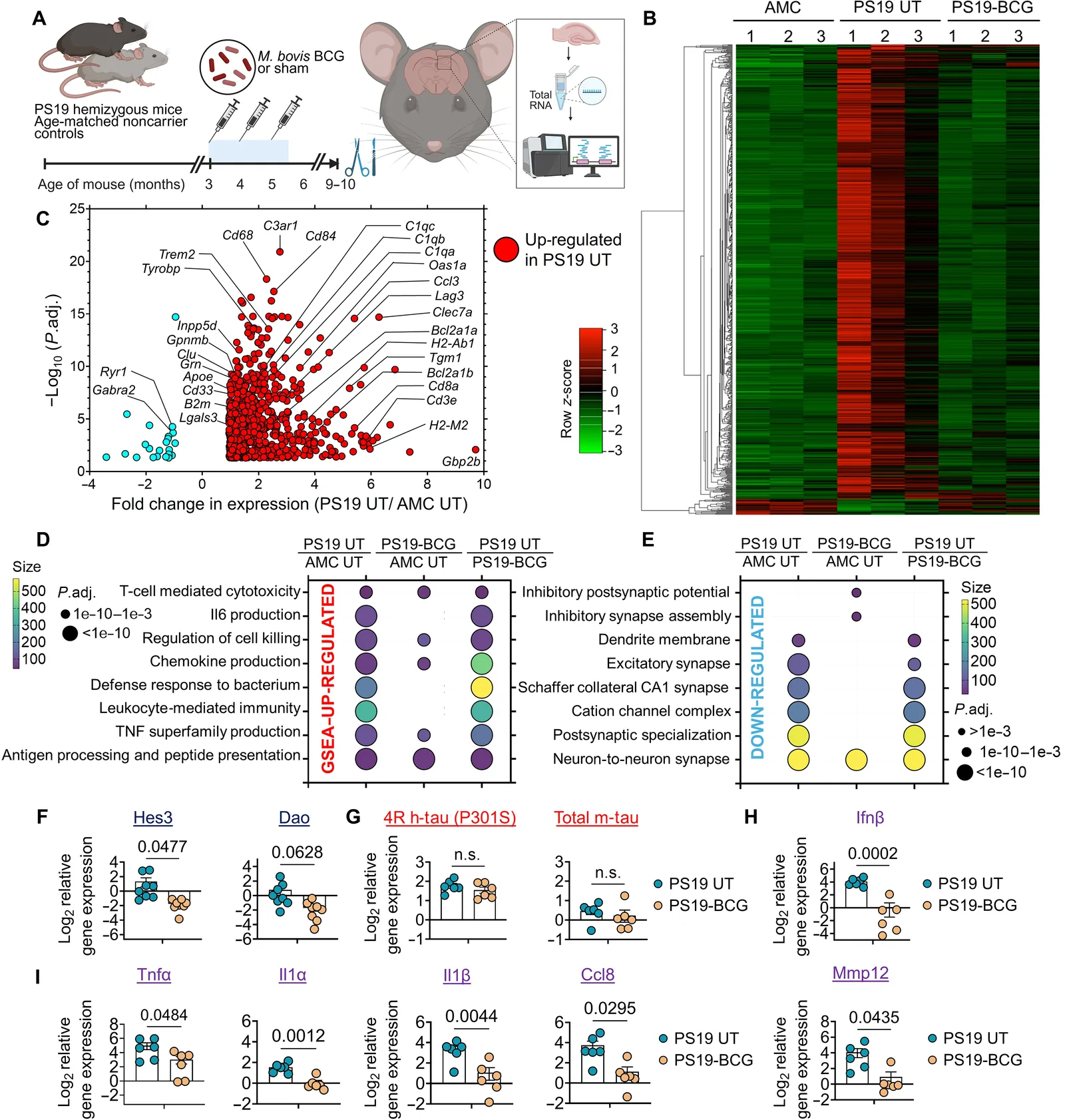
*M. bovis* BCG treatment restores homeostatic/resting transcriptional landscape in the hippocampus of PS19 mice. (**A**) Schematic of the experiment. Created in BioRender. Shee, S. (2026) https://BioRender.com/kanmk73. Bulk RNA sequencing of hippocampus tissue (*n* = 3 mice per group). (**B**) Heatmap showing transcripts per million values of all 716 differentially expressed genes (*P*.adj. < 0.05, log_2_fold change >1 or <−1) in the hippocampus from sham-treated PS19 mice relative to similarly treated age-matched noncarrier controls. Red and green color indicates up-regulated and down-regulated genes in sham-treated PS19 mice, respectively. BCG treatment restores the transcriptional landscape of PS19 mice to that of age-matched controls. (**C**) Volcano plot showing major AD-associated genes to be up-regulated (in red) in sham-treated PS19 mice relative to similarly treated age-matched noncarrier controls. GSEA revealed (**D**) topmost up-regulated pathways, and (**E**) top down-regulated pathways to be enriched under different comparisons, as indicated. Quantitative real-time polymerase chain reaction showing gene expression of (**F**) Hes3, Dao, (**G**) h-tau (P301S), total endogenous mouse tau (m-tau), and (**H**) proinflammatory cytokines relative to age-matched controls in the hippocampus. Expression of targeted genes were normalized to housekeeping gene *Gapdh*. *n* = 6 to 8 mice per group. The data are means ± SEM and representative of two independent experiments. Each data point represents a mouse. Statistical analysis between two groups was measured by unpaired two-tailed Student’s *t* test and *P* values are shown in the figure (n.s., *P* > 0.05).

In contrast, the comparison of sham-treated PS19 mice to untreated age-matched, noncarrier control revealed 716 DEGs: 692 up-regulated and 24 down-regulated genes (Fig. 5, B and C, and Supplementary data S1). In line with previously published data, up-regulated genes in untreated PS19 mice included known risk-factor genes for AD (*Apoe*, *Trem2*, *Clu*, *Tyrobp*, *Grn*, *Cd33*, *Inpp5d*, and *Oas1*). In addition to this, genes involved in complement cascades (*C1qa*, *C1qb*, *C1qc*, *C1rl*, *C3*, *C3ar1*, and *C4b*), RIG-I-like receptor signaling (*Ddx60*, *Ifih1*, *Ddx58*, *Dhx58*, *Zbp1*, *Isg15*, *Rsad2*, *Oas1b*, *Oas1g*, *Oas3*, and *Oasl1*), senescence (*40*) (*Cdkn1a*, *Bcl2a1a*, *Bcl2a1b*, *Serpine 1*, *Plaur*, *Plau*, *Cxcl16*, *Lcp1*, *Csf2rb*, *Ang*, and *Icam1*), and cytokine–cytokine receptor interaction (*Il1b*, *Il18r1*, *Il18rap*, *Tgfb1*, *Cxcl5*, *Ccl3*, *Ccl5*, *Cxcl10*, *Cxcl13*, *Cntf*, *Tnfsf13b*, *Tnf*, *Adora3*, *Csf1r*, *Cxcr3*, *Cx3cr1*, and *Il1rn*) were also up-regulated relative to age-matched controls. Moreover, functional pathway analysis by GSEA software (gene set enrichment analysis) (*41*, *42*) revealed “Antigen processing and peptide presentation” [normalized enrichment score (NES) = 2.97], “Leukocyte mediated immunity” (NES = 2.87), “TNF superfamily cytokine production” (NES = 2.88), “chemokine production” (NES = 2.84, *P*.adj. = 10^−15^), and “Il-6 production” (NES = 2.76) to be among the top 20 pathways to be enriched in sham-treated PS19 mice, clearly indicating a heightened level of inflammation (Fig. 5D). On the other hand, pathways that were significantly down-regulated in untreated PS19 mice relative to untreated AMC mice included “Neuron to neuron synapse” (NES = 2.84), “Postsynaptic specialization” (NES = 2.82), and pathways associated with neuronal homeostasis, consistent with ongoing neurodegeneration in the untreated PS19 hippocampi as seen by our previous NeuN IHC staining (Fig. 5E). Taken together, the DEGs reflect p-tau–driven neurodegeneration and inflammation in the hippocampus.

Despite the remarkable similarity between the AMC and BCG-treated PS19 transcriptome, we next asked whether we could discern any differences between the two that might provide cues as to the BCG mechanism of action. GSEA functional pathway analysis revealed “Antigen processing and peptide presentation” (NES = 2.86) and “MHC Protein complex” (NES = 2.73) to be top enriched suggesting continued innate immune cell activity in the BCG-treated PS19 mice (Fig. 5D). However, pathways involved in “cytokine or chemokine production” were relatively less enriched (*P*.adj. = 10^−5^), suggesting a dampened proinflammatory milieu compared to untreated PS19 animals. In addition, the down-regulated transcriptome in BCG-treated PS19 mice displayed a distinct pattern when compared to age-matched controls; while we still observed “neuron to neuron synapse” to be significantly down-regulated, inhibitory synapse–related gene sets emerged among the topmost down-regulated classes. These included “inhibitory postsynaptic potentials” (NES = −2), “regulation of presynaptic membrane potential” (NES = −2.02), and “inhibitory synapse assembly” (NES = −2.02; leading edge = *Cbln1*, *Gabrb3*, *Srgap2*, *Gabra6*, *Cbln4*, *Gabra2*, *Gabrb2*, *Gabra1*, *Mdga1*, *Lgi2*, *Hapln4*, *Sema4a*, *Npas4*, *Gabrg2*, and *Fgf13*, which represent genes involved in GABAergic inhibitory synapses and assembly of other inhibitory synapses) (Fig. 5E and Supplementary data S1). Taken together, the GSEA suggests a compensatory model in which untreated PS19 hippocampus is dominated by a repression of excitatory synaptic plasticity programs, while BCG treatment drives the transcriptome toward a baseline-like state characterized by down-regulation of inhibitory synaptic gene expression.

Last, we observed that the expression of three genes was uniquely repressed in BCG-treated PS19 relative to sham-treated PS19 but was unchanged in AMC relative to sham-treated PS19: Hes3 (hes family bHLH transcription factor 3; log_2_foldchange = −5.3, *P*.adj. = 0.02), Dao (d-amino acid oxidase; log_2_foldchange = −2.7, *P*.adj. = 0.04) and Gm32113 (log_2_foldchange = −5.9, *P*.adj. = 0.005) (Fig. 5F and Supplementary data S1). It has been reported that reduced levels of the *Hes3* transcriptional repressor accelerate differentiation of progenitor cells into neurons (*43*), suggesting that BCG-mediated Hes3 repression may aid in recovery from neuronal loss in the PS19 mice. In addition, d-amino acid oxidase *Dao* mediates flavin adenine dinucleotide–dependent oxidation of d-serine, d-alanine, d-proline, and d-leucine (*44*–*46*). Since d-serine is a major agonist of *N*-methyl-d-aspartate (NMDA) receptor (glutamate-gated ion channels essential for synaptic plasticity), Dao hyperactivity leads to lower d-serine levels, and consequent hypoexcitation of NMDA receptors has been linked to schizophrenia risk (*47*, *48*). We hypothesize that BCG-mediated *Dao* repression (Fig. 5F and Supplementary data S1) may increase brain d-serine availability, boosting NMDA receptor coagonist occupancy and improving synaptic plasticity and interneuron function in the PS19 mice.

Reduced p-tau levels in BCG-treated PS19 mice can be due to the repression of h-tau P301S expression (*35*). To test this, we measured mRNA levels of h-tau P301S in the hippocampus tissue of PS19 females and the age-matched controls (Fig. 5A). We observed overexpression of h-tau P301S by ≈3-fold both in sham-treated and BCG-treated PS19 mice relative to age-matched noncarrier controls (Fig. 5G). Native murine tau (m-tau) levels were also unchanged in all the experimental groups (Fig. 5G), indicating similar expression of tau in the PS19 hippocampus. In addition, bulk RNA sequencing data suggested resolution of neuroinflammation in the BCG-treated PS19 mice. To test this, we quantified the expression of proinflammatory cytokines in hippocampal RNA from BCG-treated PS19 mice, and indeed, we observed that the mRNA levels of *Ifn*β, *Tnf*α, *Il1*α, *Il1*β, *Ccl8*, and *Mmp12* were significantly reduced relative to sham-treated PS19 mice and were restored to the levels of age-matched noncarrier controls (Fig. 5, H and I).

### BCG treatment reduces the activated microglial subpopulation without altering total microglial abundance in the PS19 brain

With the above data that there is no reduction in tau gene expression, we turned to seeking mechanisms of increased clearance to explain the reduced p-tau depositions in the BCG-treated PS19 brains. A comparison of the BCG-treated PS19 versus the AMC transcriptomes by GSEA found that the top cell type–specific DEGs were as follows: ependymal cells (NES = 2.96, *P*.adj. = 3.24 × 10^−30^), dendritic cell (NES = 2.81, *P*.adj. = 2.85 × 10^−15^), macrophages (NES = 2.68, *P*.adj. = 4.7 × 10^−16^), and monocytes (NES = 2.63, *P*.adj. = 7.59 × 10^−16^). Moreover, “Antigen presentation and presentation of peptide antigen” was the top-enriched pathway (Fig. 5D and Supplementary data S1), suggesting that BCG induces a transcriptional program in myeloid cells consistent with increased capacity for uptake, lysosomal processing, and presentation of antigens, possibly tau-derived peptides, which may facilitate more efficient clearance of pathological tau aggregates.

To test this hypothesis, we isolated CD11b^+^ cells from whole mouse brains of 14-month-old sham-treated PS19, BCG-treated PS19, and age-matched control mice (Fig. 6A and fig. S4). Previous studies have reported that resting microglia under homeostatic conditions are CD11b^+^CD45^lo/int^ (*49*, *50*). However, under neuroinflammatory and neurodegenerative conditions, this tissue-resident microglial population acquires an activated CD11b^+^CD45^hi^ phenotype, making the distinction from infiltrating blood-derived/peripheral myeloid cells (CD11b^+^CD45^hi^) difficult (*49*–*51*). Therefore, to separate these populations under heightened inflammatory conditions such as those likely to be present in PS19 tauopathy, we added the CD49d marker (Integrin α4; *Itga4*) which is uniquely present in peripheral myeloid cells (*52*, *53*). With these markers, we were able to define the following brain cell populations: microglia: CD11b^+^CD49d^−^ Cd45^+(lo-hi)^, activated microglia: CD11b^+^CD49d^−^CD45^hi^, and infiltrating peripheral myeloid cells: CD11b^+^CD49d^+^CD45^hi^ (Fig. 6B and fig. S4). Gating of the CD11b^+^ parent population (and all other markers used) was established using a CD11b or respective fluorescence-minus-one (FMO) control to ensure specificity and avoid false-positive inclusion of nonmyeloid cells. Samples from two mice were pooled per measurement to ensure sufficient cell yield with a total of *n* = 6 mice per group (Fig. 6A).

**Fig. 6.**
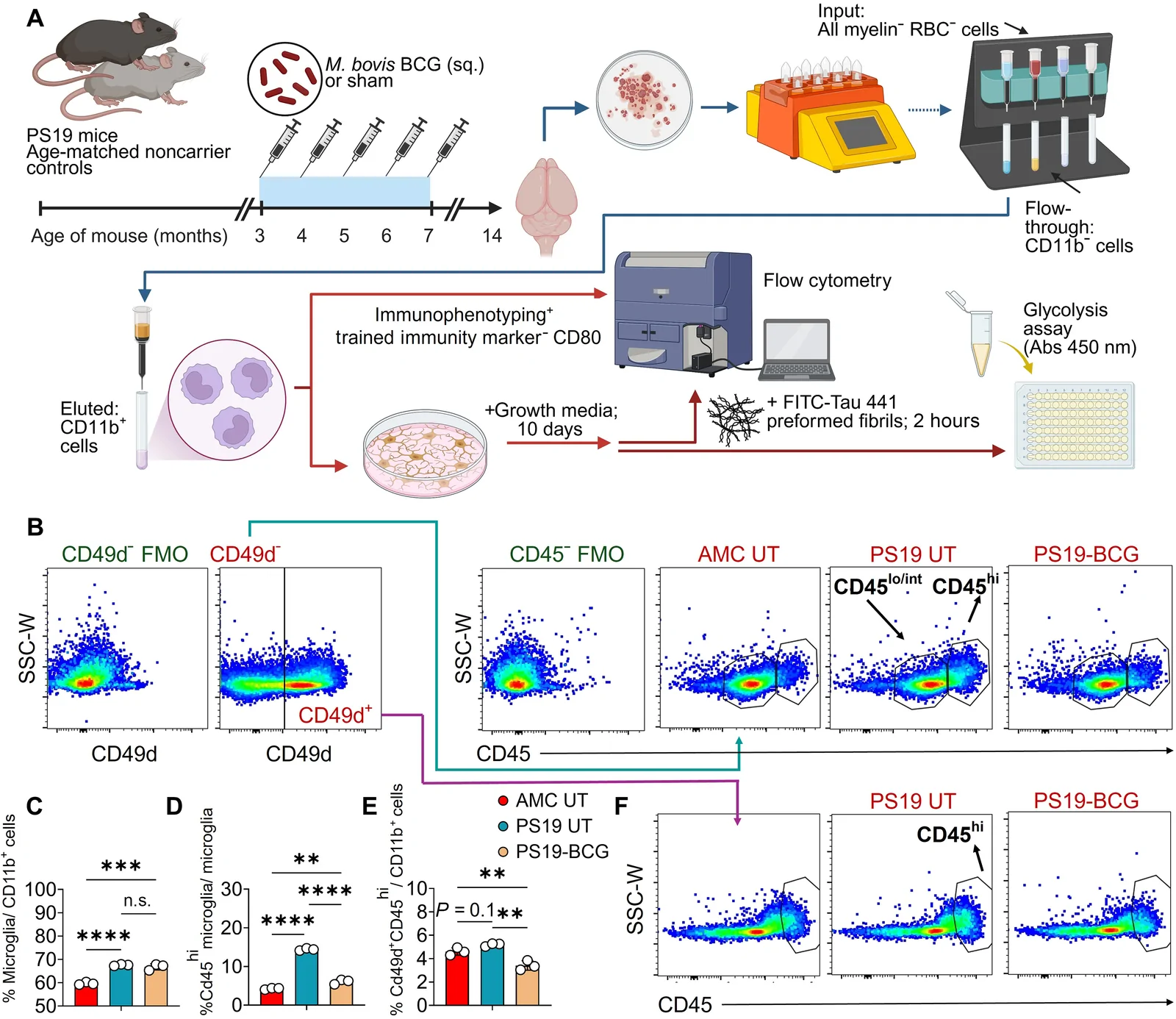
*M. bovis* BCG treatment reduces frequency of proinflammatory, activated microglial population, without reducing their total numbers in PS19 mice. (**A**) Schematic of the experiment. Created in BioRender. Shee, S. (2026) https://BioRender.com/kanmk73. Magnetic bead–based enrichment of CD11b^+^ cells from all mouse brain cells (input). CD11b-FMO (fluorescence minus one) was used to set gating of CD11b^+^ population (*n* = 6 mice per group; samples from two mice were pooled together). RBC, red blood cell. (**B**) Microglia are defined as CD11b^+^CD49d^−^Cd45^+^ (low-high expression). (**C**) %Microglial cells out of all CD11b^+^, (**D**) %activated microglia [CD45 high (hi)] out of all microglia cells, and (**E**) %peripheral myeloid cells out of all CD11b^+^ cells in age-matched controls and sham-treated and BCG-treated PS19 mice, as determined by FlowJo software. (**F**) Peripheral myeloid cells are CD11b^+^CD49d^+^Cd45high(hi). The data are means ± SEM and representative of two independent experiments. Each data point represents a mouse. Statistical analysis was calculated by one-way ANOVA with Tukey’s multiple comparisons test (n.s., *P* > 0.05; **P < 0.01; ***P < 0.001; ****P < 0.0001).

As expected, relative to age-matched controls, sham-treated PS19 mice have a significantly increased microglial population (*P* < 0.0001) (Fig. 6C) and also a higher disease-associated CD45^hi^ microglia subpopulation (CD45^hi^ out of all CD45^+^ cells = 14.4% relative to 4.2% in AMC; *P* < 0.0001) (Fig. 6D). In contrast, while we observed a similar increase in the total microglia population in the BCG-treated PS19 mice (Fig. 6C), there was significant reduction in the proportion of proinflammatory, activated microglia (CD45^hi^ out of all CD45^+^ cells = 6.04% relative to 14.4% in PS19 UT; *P* < 0.0001), indicating normalization of the microglial activation state by BCG treatment (Fig. 6D). These flow cytometry data are in agreement with the IHC data (Fig. 1C) where we observe a similar decrease in Iba1 levels in the BCG-treated PS19 hippocampus. In addition to this, the peripheral myeloid cells fraction increased in the sham-treated PS19 (5.1% out of all CD11b^+^ cells) relative to AMC (4.6%), but it was not statistically significant (*P* = 0.1) (Fig. 6, E and F). In contrast, the BCG-treated PS19 brains exhibited decreased infiltration of peripheral myeloid cells (3.4%), probably because of dampened neuroinflammation. Together, these data suggest that BCG therapy recalibrates the microglial composition in the brain—maintaining high microglial abundance while selectively dampening their proinflammatory state—thus reducing tissue-damaging inflammation (Fig. 5, H and I) yet preserving a workforce to remove p-tau deposits.

### BCG therapy induces trained-immunity features in brain myeloid cells

We hypothesized that AD prevention after BCG therapy may share features with BCG-mediated myeloid cell “trained immunity” (*15*–*17*). Previous studies have established that BCG exposure “trains” central as well as peripheral immune cells by inducing both epigenetic (H3K4me3 and H3K27ac activation marks on immune-associated genes), metabolomic changes (increased glycolysis) and up-regulation of pattern recognition receptors (*Tlr*), and costimulatory molecules (CD80 and CD86) (*54*–*56*). The “training” occurs after the cell encounters a first stimulus (BCG in our study) and leads the cell to then display an elevated response against an unrelated, heterologous second stimulus (p-tau deposits in our study). To test this hypothesis, we measured the median fluorescence intensity of the CD80 surface marker on microglial and peripheral myeloid cells. In addition, we quantified the phagocytosis and glycolysis levels of isolated CD11b^+^ cells (*57*, *58*). The CD80 levels on both microglial and peripheral myeloid cells from sham-treated PS19 mice were higher relative to age-matched controls (Fig. 7, A and B), probably because of heightened inflammation. We observed higher expression of CD80 on both myeloid cell types, but the peripheral myeloid cells from the BCG-treated PS19 mice exhibited increased expression of CD80 relative to both sham-treated PS19 and age-matched controls (*P* < 0.05), suggesting compartment-specific induction of trained immunity (Fig. 7, A and B).

**Fig. 7.**
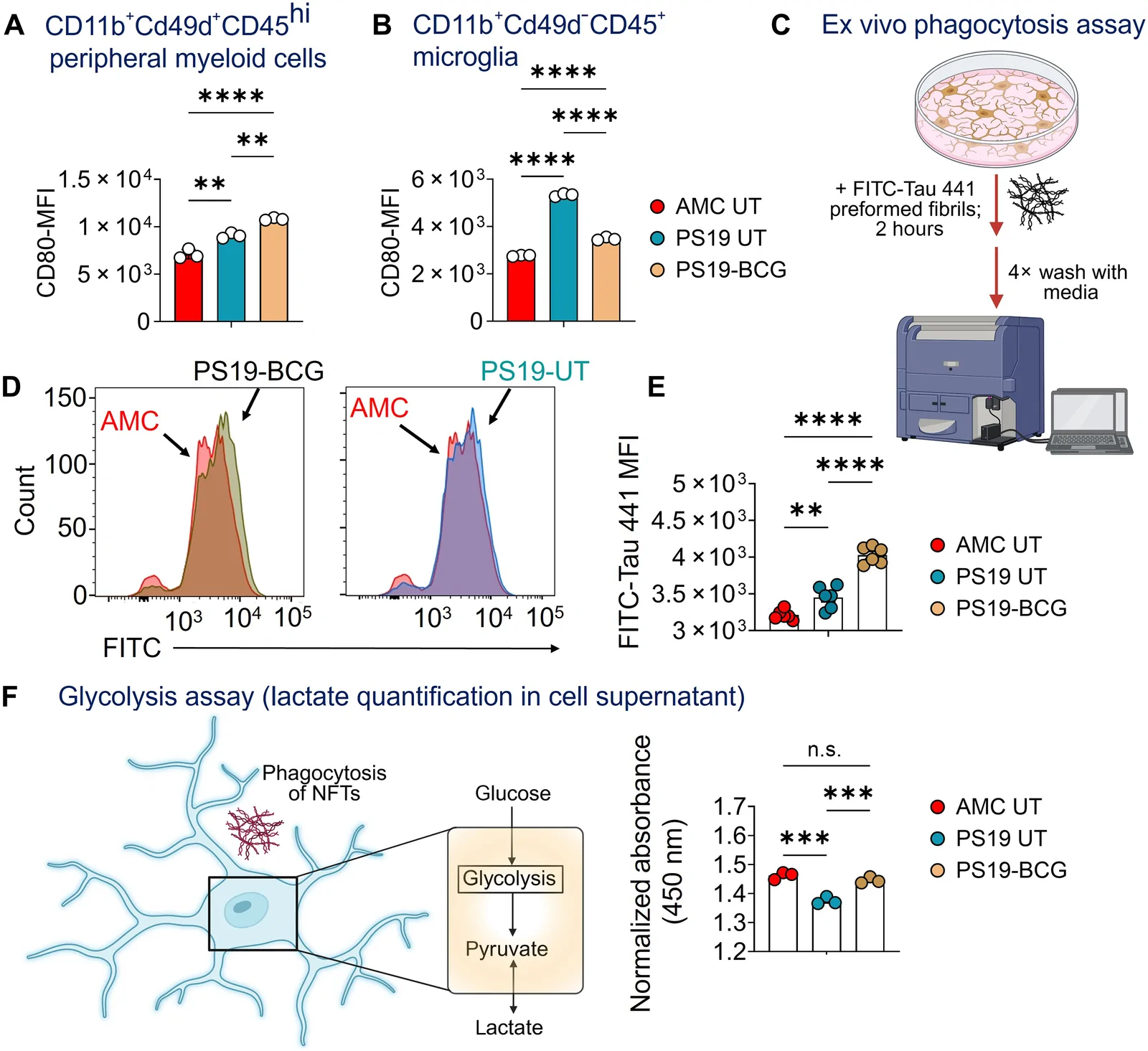
CD11b^+^ myeloid cells from BCG-treated PS19 mice brain displayed significantly higher markers of trained immunity. Median fluorescence intensity (MFI) of the surface marker of trained immunity, CD80, on (**A**) peripheral myeloid cells and (**B**) microglia, as determined by FlowJo software (*n* = 6 mice per group; samples from two mice were pooled together). (**C**) Schematic of the phagocytosis assay with primary CD11b^+^ cells. (**D**) Histogram showing FITC-tau uptake by microglia and respective (**E**) quantification. (**F**) Lactate concentration in the supernatant of resting CD11b^+^ cells, as determined by lactate dehydrogenase–based colorimetric assay. The absorbance at 450 nm was normalized to respective protein levels. The data are means ± SEM and representative of two independent experiments. Statistical analysis was calculated by one-way ANOVA with Tukey’s multiple comparisons test (n.s., *P* > 0.05; **P < 0.01; ***P < 0.001; ****P < 0.0001). Schematics were created in BioRender. Shee, S. (2026) https://BioRender.com/kanmk73.

Next, we measured the phagocytic capacity of primary brain CD11b^+^ cells ex vivo, using a physiologically relevant substrate, fluorescein isothiocyanate (FITC)–conjugated h-tau–441 fibrils. Adherent, primary brain CD11b^+^myeloid cells were expanded in growth media for 10 days. They were then coincubated with FITC–h-tau fibrils for 2 hours, extensively washed to remove extracellular fibrils, and then subjected to flow cytometry (Fig. 7C). While we observed a significantly high uptake of tau fibrils by CD11b^+^ cells from sham-treated PS19 mice (7.5% higher relative to AMC; *P* = 0.007), the CD11b^+^ cells from BCG-treated PS19 mice exhibited the highest uptake of the fibrils (25.4% higher relative to AMC; *P* < 0.0001) (Fig. 7, D and E). These data indicate that BCG-trained brain myeloid cells have an enhanced capacity for phagocytosis of p-tau–related protein deposits. In agreement with trained phenotype, CD11b^+^ brain myeloid cells from BCG-treated PS19 displayed higher production of lactate relative to sham-treated PS19 mice (Fig. 7F), suggesting an elevated glycolysis setpoint at their baseline/resting state.

In sum, our results suggest that BCG therapy in the PS19 model shows features of myeloid cell immune training and that BCG therapy restricts microglial hyperactivation/microgliosis, while priming peripheral and/or brain myeloid cells for heightened p-tau–NFT phagocytosis, thereby mitigating tauopathy in the PS19 mice (Fig. 8).

**Fig. 8.**
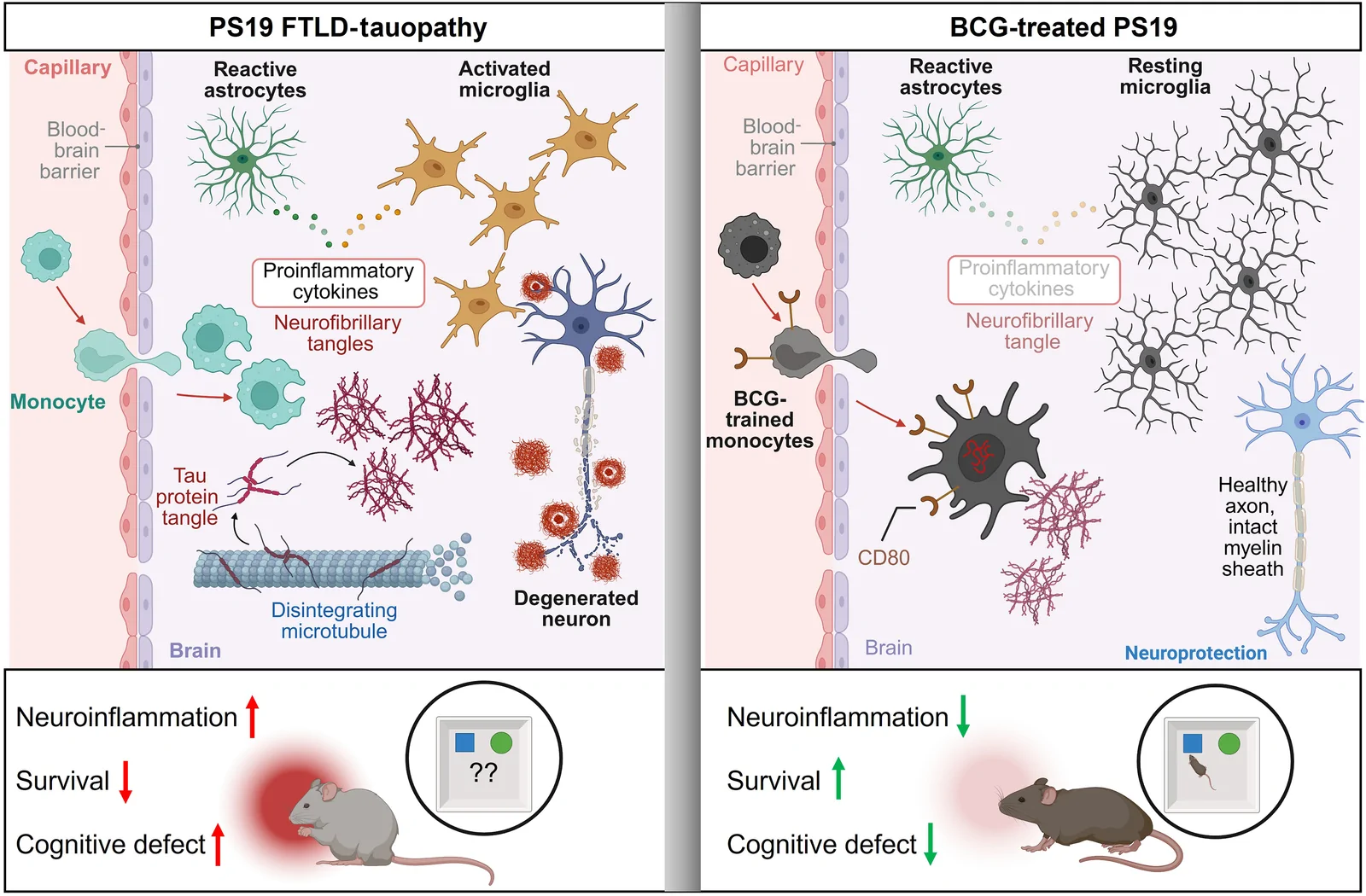
BCG-trained myeloid cells reduce neuroinflammation and neurodegeneration in PS19 tauopathy mice. (Left) PS19 mice brains display blood-brain barrier dysfunction with infiltrating monocytes, reactive astrocytes, and activated microglia that release proinflammatory cytokines. Pathology includes tau aggregation into NFTs and neuronal degeneration, accompanied by heightened systemic inflammation and cognitive/behavioral deficits. (Right) BCG treatment induces trained monocytes (CD80^+^), which traffic to the brain, phagocytose and degrade NFTs, dampen microglial activation, and reduce proinflammatory cytokines. This is associated with fewer p-tau tangles, preserved axons and myelin, and overall neuroprotection, leading to reduced inflammation and improved cognitive performance in PS19 mice. Created in BioRender. Shee, S. (2026) https://BioRender.com/kanmk73.

## DISCUSSION

Our study aims to assess BCG in a dementia model of tau pathology and to identify mechanisms by which BCG may promote clearance of pathologic tau NFTs. We found that early BCG administration starting at 3 months of age reduced brain atrophy, p-tau levels, and neuronal loss measured at the later time point of 8 to 9 months. Simultaneously, BCG therapy restored glutamate levels in vivo and improved recognition-memory performance, body weight, and survival of PS19 mice, suggesting key disease modifying effects.

One of the most remarkable effects of BCG that we observed in this study came from RNA sequencing of the hippocampus of 9.5-month-old mice revealing restoration of the transcriptomic landscape to near normal levels in BCG-treated PS19 mice (Fig. 5B); this effect was mediated by an intervention that was both physically remote (hypodermis to brain) and temporally remote (4.5-month time interval). Our RNA-sequencing analysis provided a valuable overview of BCG-mediated changes on the hippocampal transcriptional landscape; moreover, it served as a useful hypothesis generating tool, leading us to several testable postulates such as: (i) whether BCG treatment of PS19 mice may stimulate neuronal survival by down-regulating inhibitory synaptic gene expression, (ii) whether BCG may promote neuronal differentiation via down-regulation of *Hes3*, or (iii) whether BCG may boost NMDA synaptic signaling via *Dao* down-regulation and increased d-serine availability. Autophagy has been shown to be an important mechanism underlying the BCG-induced trained immunity (*59*, *60*). Therefore, it is possible that BCG-mediated induction of autophagy might be significant in the context of tauopathy; however, we have not tested this in our study. GSEA of the transcriptome data (Fig. 5, A and B) did not detect pathways associated with autophagy to be deregulated in BCG-vaccinated PS19 mice in this experimental setting.

Although large-scale prospective studies have yet to show definitively whether BCG does or does not prevent dementia, there has been literature speculation around potential mechanisms of BCG neuroprotection. These include (i) central nervous system (CNS) anti-inflammatory activity, (ii) immunometabolic reprogramming, (iii) epigenetic reprogramming, (iv) boosting host antimicrobial defense against heterologous pathogens such as HSV-1 or *Porphyromonas gingivalis* that might drive or contribute to dementia risk (*61*). The latter three mechanisms are each properties associated with BCG-mediated trained immunity and can be readily demonstrated with in vitro systems (*62*, *63*), animal models (*64*), and human BCG intervention studies (*65*).

Mechanistically, our study identified BCG-induced trained immunity properties as playing key roles in the clearance of tau NFTs. Using the surface costimulatory molecule CD80 as a marker of trained immunity, we found that BCG vaccination led to a significant increase in the recruitment of CD80^+^ peripheral myeloid cells to the brain where they are likely to play a role in removal of NFT debris. In addition, we found that adherent CD11b^+^myeloid cells from BCG-treated PS19 mice were significantly more active in phagocytosis of h-tau–441 fibrils and in the generation of lactate (glycolytic metabolomic shift), both known traits of trained immune myeloid cells. Last, we found that BCG mechanistically reduces the inflammatory state of brain microglial cells. While both treated and untreated PS19 mice had significantly elevated populations of brain microglial cells compared to healthy AMC mice, a much higher proportion of those microglial cells were activated in untreated PS19 mice (microgliosis) than in BCG-treated PS19 mice. The mechanisms of BCG action in the PS19 model are summarized diagrammatically in Fig. 8. Furthermore, BCG-induced trained immunity is known to be mediated by epigenetic reprogramming through H3K4me3 and H3K27Ac histone modifications at several loci related to innate immunity (*66*). Therefore, future experiments using chromatin immunoprecipitation sequencing and single-nucleus RNA sequencing will be necessary to elucidate the molecular mechanisms and identify potential endogenous mediators.

While our study focuses on addressing BCG in a tauopathy model of dementia, others have tested the efficacy of BCG for AD in the APP/PS1 β amyloid (Aβ) mouse model and demonstrated improved cognition in BCG-treated APP/PS1 mice compared to untreated animals (*67*–*69*). Across these three studies, there were confounding results on the level of reduction (or lack thereof) of Aβ plaque formation, CNS anti-inflammatory cytokine levels [interleukin-4 (IL-4), IL-10, or transforming growth factor–β], and neurotrophic factors (brain-derived neurotrophic factor). Recruitment of IL-10–producing monocytes were shown to increase in the areas of Aβ plaques (*67*–*69*). A concern with these studies is that brain atrophy correlates closely with p-tau accumulation and less with Aβ deposition (*20*, *70*–*72*). Our study also has certain limitations. The p-tau–driven neurodegeneration observed in the PS19 model most closely parallels the human dementia syndrome of frontotemporal lobar degeneration with tau pathology rather than classical human AD, which also includes Aβ deposition. The sex differences observed in the PS19 model (females survive longer) are also a limitation; it is not feasible to compare sex- and age-matched animals since the disease is far more advanced in males at any given time point. An additional limitation of this study is that it was performed solely with BCG administered via the subcutaneous route, although TB prevention animal model studies have shown that the intravenous route confers superior protective efficacy (*73*–*75*). Our group intends to evaluate alternative routes of BCG administration in future studies.

There is an urgent need for AD-modifying drugs since the three Food and Drug Administration–approved agents currently slow but do not stop dementia progression, and neither cure nor proven prevention has been achieved (*76*–*79*). AD belongs to the broader class of tauopathy syndromes that are characterized by the progressive accumulation and spread of misfolded, hyperphosphorylated tau fibrils leading to synaptic failure, neuronal death, and cognitive decline (*80*). Histologically, AD also involves Aβ plaque deposition that precedes tau aggregation (*81*, *82*). Given the failure of antiamyloid therapies in clinical trials and the strong clinical correlation between NFT accumulation with AD severity in humans (*70*, *83*–*86*), novel strategies ameliorating tauopathy may mitigate AD onset.

BCG is a live attenuated bacterial strain developed by serial passage of *Mycobacterium bovis* from 1908 to 1921 (*87*) and may be the most widely administered vaccine in human history, with an estimated 290 million doses administered to newborns annually to prevent TB (*88*–*91*). BCG is also a critical immunotherapy for bladder cancer and is considered first-line therapy against NMIBC, which is the most common form of bladder cancer with an incidence of ∼61,500 cases per year in the Unites States (*17*, *92*). BCG is also considered one of the world’s safest vaccines. Because of over one century of human use, adverse effects (AEs) of BCG are exceptionally well defined. Local injection site reactions (pain and swelling) and flu-like symptoms occur at rates comparable to other vaccines (*93*). Severe AEs—disseminated BCG infection (0.1 to 0.2 per 100,000) (*94*) and osteitis (0.3 to 1.0 per 100,000) (*95*)—are very rare.

While other vaccines such as the shingles vaccines, Zostavax and Shingrix, may also have dementia-preventative activity, these and most other vaccines that may have dementia-protective activity are already in use in the United States and most developed countries. Uniquely, BCG is a safe, readily manufactured vaccine that is not used in the United States, Canada, Australia, New Zealand, and most western European nations (because of a low incidence of TB)—the very same populations that bear the highest burden of reported AD and related dementias. Our study strongly suggests that dementia protection by BCG is a credible observation that merits future basic research and large-scale prospective clinical trials to provide definitive answers.

## MATERIALS AND METHODS

### Animals used in this study

The PS19 mouse line [B6;C3-Tg(Prnp-MAPT*P301S)PS19Vle/J] were purchased from the Jackson Laboratory [6 weeks old; strain: no. 008169 (Bar Harbor, ME, USA)]. This mouse strain was bred and maintained at the Rodent Vivarium of Johns Hopkins University, School of Medicine and was used in this study. The breeding strategy was to mate noncarrier × hemizygote, and genetic identity was confirmed by polymerase chain reaction (PCR)–based genotyping. The oligonucleotides used for the PCR are shown in Supplementary data S2. All noncarrier age-matched controls used were littermates of hemizygous PS19 mice. All experiments were conducted under protocols approved by the Johns Hopkins Animal Care and Use Committee (protocol no.: MO23M47) and followed established ethical animal handling and care guidelines. Animals were housed in individually ventilated cages under controlled conditions, including a 12-hour light/dark cycle, ambient temperatures ranging from 20° to 24°C, and relative humidity between 45 and 65%. Each cage contained no more than five mice of the same strain/sex to ensure proper animal welfare. Mice were randomly assigned to experimental groups to minimize bias. Humane and painless euthanasia procedures were used in compliance with AVMA (American Veterinary Medical Association) guidelines.

All experiments involving *M. bovis* BCG were conducted in biosafety level 2 facilities at Johns Hopkins University, School of Medicine, following protocols approved by the Institutional Biosafety Committee. Mice were briefly restrained during subcutaneous injection to ensure accurate delivery. All animals were monitored for signs of illness, such as weight loss or lethargy, and those that became moribund before the experiment’s conclusion were promptly euthanized, except in survival studies.

### Bacterial culture and subcutaneous administration into mice

*M. bovis* BCG Pasteur TMC 1173P2 was obtained from the Johns Hopkins University, Center for Tuberculosis Research. BCG was grown on 7H11 agar (Becton, Dickinson and Company, USA) supplemented with OADC (BD; 212351) at 37°C. CFU enumeration was performed by plating serial dilutions on 7H11 agar supplemented with OADC, followed by incubation at 37°C for 3 to 4 weeks.

For subcutaneous vaccination of mice, BCG cryostocks in Freezing mix (7H9 +15% glycerol) (optical density = 1.2) were thawed, cells were washed twice with sterile 1× PBS and resuspended in 1× PBS. BCG strain was administered into mouse in a total volume of 0.2 ml per treatment. PS19 mice were either injected with PBS (1× PBS; sham) or BCG Pasteur strain (2 × 10^6^ CFU) subcutaneously into the dorsal neck (scruff) region of mice. All mice were monitored daily for signs of tauopathy progression, including weight loss, paresis, lethargy, hunched posture, and reduced activity. Survival data were analyzed using Kaplan-Meier survival curves to determine the median time to death and assess differences between treatment groups.

### IHC against p-tau, Iba1, NeuN, and GFAP

At indicated time points, the mice were euthanized by isoflurane (VetOne, Fluriso, MWI Animal Health, Boise, ID, USA) overdose. The mouse brains were harvested after transcardial perfusion with ≈15 ml of ice-cold 1× PBS (Gibco, catalog no. 10010) and dissected on a chilled surface into two halves from midline. The left brain was dissected into hippocampus and rest of the brain, flash frozen in liquid nitrogen, and stored at −80°C, until tissue processing. The right half of the brain was fixed in 4% paraformaldehyde solution [paraformaldehyde (Sigma-Aldrich, catalog no. P6148) in 1× PBS] for at least 24 hours at 4°C, before it was submitted to the SKCCC Oncology Tissue and Imaging Service Core Laboratory (Johns Hopkins University, School of Medicine) for downstream processing. The brains were paraffin embedded and 10 μm sectioned.

For IHC, the slides were deparaffinized, and antigen retrieval was performed using 10 mM sodium citrate buffer (pH = 6), to efficiently expose the epitope to the antibodies. Nonspecific binding of antibodies was eliminated by incubating with blocking buffer [1.5% normal goat serum (Vector Laboratories, catalog no. S-1000-20) in PBS with 0.1% Triton X-100] for 1 hour. Slides were then incubated in 0.3% H_2_O_2_ (Sigma-Aldrich, catalog no. H1009) for 30 min to quench endogenous peroxidase. Primary antibodies were prepared in blocking buffer and applied overnight at room temperature followed by incubation with biotinylated secondary antibody (Vector Laboratories, Anti-mouse Ref. BP-9200-50; Anti-rabbit Ref. BP-9100-50) for 1 hour. Peroxidase-labeled ABC reagent (Vector Laboratories, Newark, CA, USA) was applied for 30 min followed by signal development using 3,3 diaminobenzidine (DAB) (Vector Laboratories). Slides were counterstained with hematoxylin, dehydrated, cleared, and mounted. The following primary antibodies were used: antiserum against NeuN (1:1000; MAB377, Merck), p-tau AT8 (1:1000; MN1020, Invitrogen), GFAP (1:1000; Z0334, Dako Cooperation), and Iba1 [1:1000; EPR16588 (ab178846), Abcam]. Manufacturer-validated antibodies with demonstrated specificity were used. For each mouse brain sample, IHC was done in three planes (10-μm-thick sections) for all the antibodies.

Slides were digitally scanned at 40× using a Hamamatsu Nanozoomer S210 digital slide scanner (Hamamatsu Photonics, Shizuoka, Japan) at ×40 magnification (0.23 μm per pixel). Image files were submitted in https://digital.pathology.johnshopkins.edu/ using the Concentriq LS platform (Proscia, PA, USA). %DAB-positive area was quantified as a percentage of the total region of interest, using an in-house protocol (fig. S1) developed with ImageJ software version 1.54m (NIH, USA) to minimize human bias, through standardized software-based settings.

### Behavioral tests

Behavioral assessments commenced following 5 months of vehicle or BCG vaccination treatment (*n* = 8 mice per group). Female mice were transferred to a reversed light cycle room (12:12-hour dark:light) 2 weeks before testing to allow circadian rhythm adaptation. All tests were conducted during the dark phase under dim red lighting. To minimize cumulative stress and carryover effects, only one test was performed per day, arranged in order of increasing stress intensity: OFT and NORT. Before each test, mice were acclimatized for 30 min in their home cages within the testing room. All apparatus were thoroughly cleaned with Vimoba disinfectant before each session and between animals to eliminate residual odor cues.

### Open-field test

The OFT was conducted to assess locomotor activity. Mice were individually placed in the center of a square open-field arena (40 cm by 40 cm by 40 cm) and allowed to explore freely for 30 min. Locomotor activity was recorded using a San Diego Instruments Photobeam Activity System–Open Field (PAS-Open Field), equipped with a 16 by 16 photobeam frame with 1-inch (2.54-cm) beam spacing surrounding the enclosure. Parameters measured included total beam breaks as an index of general locomotor activity.

### Novel object recognition test

The NORT was conducted to assess recognition memory. The test was performed in an open-field arena (40 cm by 40 cm by 40 cm) over two consecutive days, consisting of three phases: habituation, training, and testing. On the first day (habituation phase), mice were placed individually into the testing chamber without objects and allowed to explore freely for 10 min. After 24 hours, the training phase was conducted, in which two identical objects (matched for color, size, material, and shape) were placed along the diagonal of the chamber. Mice were allowed to explore both objects freely for 10 min. Following a 60-min intertrial interval, the test phase was conducted, in which one familiar object was replaced with a novel object that differed only in shape. Mice were allowed to explore freely for 5 min. The position of the novel object was counterbalanced across subjects to avoid location preference bias. Animal behavior was recorded using an overhead video camera, and object exploration duration was quantified by the blinded observers. The recognition index was calculated as the proportion of novel object exploration time relative to total exploration time. The discrimination index was calculated as: (Novel object exploration time − Familiar object exploration time) / Total exploration time.

### T2-weighted MRI

Acquisition: MRI experiments were conducted on a simultaneous 7 T/30 PET/MRI scanner (Bruker BioSpin) equipped with a 72-mm inner-diameter transmit/receive volume coil and a 1H receive-only mouse brain surface coil. Mice were anesthetized with isoflurane and maintained under anesthesia throughout image acquisition with continuous respiratory monitoring. After the localizer, anatomical brain MRI scans were acquired using an axial 2D TurboRARE T2-weighted sequence with the following parameters: repetition time = 4000 ms, echo time = 20 ms, field of view = 22 by 22 mm^2^, matrix size = 256 by 256, which yielded in-plane resolution 0.086 by 0.086 mm^2^ resolution, slice thickness was 0.3 mm with 12 signal averages.

Analysis: After acquisition, DICOM images were analyzed using either ITK-SNAP 4.4.0 software (*36*) or by VivoQuant 2020 (Invicro). With VivoQuant 2020, a semiautomatic segmentation method was used to quantify ventricular and hippocampal volumes with the 3D Brain Atlas Tool. Ventricular CSF was segmented using a predefined threshold, while the hippocampus was delineated using an automatic anatomic segmentation tool. All segmentations were manually refined with reference to a digital mouse brain atlas. Three-dimensional models were then generated, and total ventricular and hippocampal volumes were calculated and recorded. Investigators were blinded to study group allocation during image analysis.

### Glutamate-weighted CEST-MRI

CEST MRI experiments were performed using a continuous-wave saturation pulse (1 s long, 3 μT in amplitude) followed by a fast spin-echo MRI readout. A total of 61 saturation frequency offsets were acquired between ±6 parts per million (ppm) (equally spaced). An additional acquisition at 50 ppm was used for normalization. In-plane field of view was 22 by 22 mm^2^ with a matrix size of 64 by 64 (in-plane resolution 0.34 by 0.34 mm^2^), and slice thickness was 1.5 mm. Data were processed by first correcting for B_0_ field inhomogeneity by voxel-wise fitting of the bulk water peak and assigning it as 0 ppm. The amplitude of the glutamate peak at 3 ppm was mapped on a voxel-wise basis using MTRasym analysis (*96*) and then taking the mean MTRasym signal between 3.5 and 2.5 ppm. All data were analyzed in python using in-house written scripts.

### Bulk RNA sequencing of mouse hippocampus

RNA was extracted from hippocampus tissue samples using the RNeasy Mini kit (Qiagen, catalog no. 74104) and quantity and quality assessed by Bioanalyzer (Agilent Technologies, Palo Alto, CA, USA). Depletion of ribosomal RNA was performed using the Qiagen Fast Select rRNA HMR kit (Qiagen, catalog no. 334387) according to the manufacturer’s instructions. A stranded mRNA library was prepared using the Illumina TruSeq Stranded Total RNA kit (Illumina, catalog no. 20020596) according to the manufacturer’s instructions. Sequencing was performed using the NovaSeq 6000 X platform sequencer and the TruSeq Cluster Kit (Illumina), resulting in approximately 50 million paired-end reads per sample. The samples were sequenced using a 2 × 150 paired-end configuration. Illumina’s bcl2fastq v2.20.0 was used to convert BCL files to FASTQ files. Raw sequencing quality was assessed using FastQC v0.11.7, and adapter trimming and quality filtering were performed with fastp v0.24.0. The processed reads were aligned to the mouse reference genome (mm39) using RSEM v1.3.3, which generated BAM files along with gene and transcript level expression estimates. Differential expression analysis and principal components analysis were conducted using DESeq2 v1.44.0. GSEA was performed with fgsea v1.30.0, using gene sets obtained from the Molecular Signatures Database. Raw data were deposited in NCBI’s Gene Expression Omnibus (GEO, accession no.: GSE326626; www.ncbi.nlm.nih.gov/geo/query/acc.cgi?acc=GSE326626).

### Quantitative real-time PCR from mouse hippocampus

At the indicated time point, mouse hippocampus tissue was lysed in TRIzol (Thermo Fisher Scientific; 15596026), and RNA was extracted using the Qiagen RNeasy Mini Kit (74104) and the RNase-free DNase Set (Qiagen; 79254), following the manufacturer’s protocol. RNA purity and concentration were quantified using NanoDrop One (Thermo Fisher Scientific). RNA (1 μg) was used for cDNA synthesis using the iScript cDNA Synthesis Kit (Bio-Rad; 1708891) and quantitative real-time PCR was done with QuantStudio 3 (Applied Biosystems; Thermo Fisher Scientific) using gene-specific primers (Supplementary data S2) and iTaq Universal SYBR Green Supermix (Bio-Rad; 1725121). Expression of genes was normalized with Ct value for *Gapdh* expression (internal housekeeping control).

### Isolation of CD11b^+^ cells from mouse brain

An adult brain dissociation kit (Miltenyi Biotec, catalog no. 130-107-677) was used to dissociate brain into single-cell suspensions, following the manufacturer’s instructions. Briefly, the mice were euthanized via isoflurane overdose; their brains were harvested following complete transcardial perfusion with 15 ml of chilled 1× D-PBS (Thermo Fisher Scientific, catalog no. 14287072), washed in D-PBS, cut into small pieces using scalpel, and collected in 1950 μl of Enzyme mix 1 + 30 μl of Enzyme Mix 2 containing gentleMACS C-tubes. The cut tissue was dissociated using the gentleMACS Octo Dissociator (program- 37C_ABDK_01), and single-cell suspensions were obtained by filtering dissociated tissue through 100-μm MACS Smartstrainers (Miltenyi Biotec, catalog no. 130-110-916). This was followed by removal of myelin debris and red blood cells, following the manufacturer’s protocol. All calculations were based on the original mass of brain = 400 to 500 mg. Single cells were collected in PB buffer [D-PBS + 0.5% fetal bovine serum (MilliporeSigma, catalog no. F4135)], passed through the 70-μm MACS Smartstrainer (Miltenyi Biotec, catalog no. 130-098-462) followed by centrifugation for 10 min at 4°C, 350*g*. The strainer was prerinsed with buffer to maximize recovery. The cell pellets were gently resuspended in 90 μl of ice-cold PB buffer per 1 × 10^7^ total cells by gentle pipetting, as recommended by the manufacturer. CD11b magnetic MicroBeads (Miltenyi Biotec, 130-093-634) were added at 10 μl per 1 × 10^7^ cells, mixed by gentle inversion, and incubated for 15 min at 2° to 8°C in the dark. The cells were washed with 1 ml of ice-cold PB buffer and centrifuged at 300*g* for 5 min; supernatants were aspirated completely. Pellets were resuspended in 1000 μl of PB buffer, and 20 μl of the suspension was reserved for subsequent flow cytometric analysis as the input (pre-enrichment) fraction. For magnetic separation, LS columns (Miltenyi Biotec, 130-042-401) with prerinsed 70-μm preseparation filter were placed in a QuadroMACS Separator. Columns were equilibrated with 3 ml of PB buffer before sample loading. The labeled cell suspension was applied to the column, and the flow-through was discarded. Columns were washed with PB buffer (three times with 3 ml), removed from the magnetic field, placed over a clean collection tube, and magnetically retained cells were eluted with a plunger to obtain the CD11b^+^ fraction in PB buffer. Both fractions were centrifuged at 300*g* for 10 min at room temperature, supernatants were completely aspirated, and pellets were resuspended in 1 ml of PB buffer. These cells were used immediately for downstream assays.

### Immunoprofiling of brain tissue by multicolor flow cytometry

Primary CD11b^+^ cells were counted by a cell counter (Invitrogen Countess Automated Cell Counter; C10281). We isolated ≈ 2 × 10^5^ CD11b^+^ cells per mouse brain, and samples from two mouse samples were pooled together. The cells were centrifuged at 300*g* for 5 min, supernatant was aspirated out completely and resuspended in 1× PBS. Primary cells were stained for viability using a live-dead near-infrared (IR) dye (1:1000 dilution in PBS; Thermo Fisher Scientific; L10119) and blocked with Rat Anti-Mouse CD16/CD32 Fc Block (Clone 2.4G2, BD Pharmingen; 553142). The panel of antibodies was designed using FluoroFinder (https://fluorofinder.com/). For extracellular staining, cells were incubated with 50 μl of an antibody cocktail in Brilliant Stain Buffer (BD Biosciences; 563794) containing anti-mouse CD45 (Brilliant Violet 480; BD Biosciences; 566095), CD11b (Brilliant Violet 650; BioLegend; 101259), CD49d (allophycocyanin; BioLegend; 103622), CD80 (B7-1) monoclonal antibody (16-10A1; eFluor 450; eBioscience; 48-0801-82) for 30 min at 4°C in the dark. After staining, cells were fixed with 4% paraformaldehyde solution, washed with cell staining buffer (BioLegend; 420201), filtered through strainer-capped BD FACS tubes (BD; 352235), and analyzed via flow cytometry. A total of 1 × 10^5^ events per sample were acquired using a BD LSRFortessa Cell Analyzer (BD Biosciences), and the data were analyzed with FlowJo software version 10.8.1 (BD Biosciences). Single-stain controls, FMOs, and compensation matrices were prepared using cells. All flow antibodies were titrated to determine optimal staining concentrations, and only manufacturer-validated antibodies with demonstrated specificity were used. For the analysis, cells were first gated on singlets (FSC-H versus FSC-A) and live cells (live-dead near-IR negative) before further selection of cell types. Microglia and peripheral myeloid cells were defined as follows: Cd11b^+^Cd45^+^CD49d^−^ and Cd11b^+^Cd45^hi^CD49d^+^ cells, respectively. Gating for all the positively stained events were established on the basis of respective FMO and unstained controls.

### Primary cells culture and phagocytosis assay

Primary CD11b^+^ cells were isolated from brain tissue as described above. Single cells were seeded at a density of 5 × 10^4^ cells per well onto poly-d-lysine (PDL) (Sigma-Aldrich; A003E)–coated 24-well plates in Dulbecco’s modified Eagle’s medium (high glucose, GlutaMAX; Thermo Fisher Scientific; 10566016) containing 10% heat-inactivated fetal bovine serum (MilliporeSigma; F4135), 1× antibiotic-antimycotic solution (Sigma-Aldrich; A5955), and macrophage colony-stimulating factor (100 ng/ml) (PeproTech, catalog no. 315-02). The cells were maintained at 37°C in a humidified incubator with 5% CO_2_ for 10 days (*97*). The following day, medium was replaced, and cells were treated with 5 μg of FITC-conjugated h-tau–441 preformed fibrils (rPeptide; TF-1113-2) for 2 hours at 37°C in a humidified atmosphere with 5% CO_2_. After treatment, cells were washed twice with PBS and scraped out in 1 ml of RPMI 1640 (without phenol red) (Gibco; 11835). Live-dead Fixable Near-IR Dead Cell Stain (Thermo Fisher Scientific; L10119) was added for the detection of dead cells. Flow cytometry was immediately done using a CytoFLEX cytometer (Beckman Coulter), and 10,000 events per sample were recorded. The FlowJo software was applied for the quantification of cell population and fluorescence intensity.

### Glycolysis assay

The glycolysis capacity of CD11b^+^ cells were measured using the Glycolysis/OXPHOS Assay Kit (Dojindo, Tabaru, Japan; G270) according to the manufacturer’s instructions. Single cells were seeded at a density of 10^4^ cells per well onto PDL-coated 96-well plates and maintained at 37°C in a humidified incubator with 5% CO_2_ for 10 days in complete media. The following day, medium was replaced with fresh media and incubated further for 2 days. The lactate in the culture supernatant was quantified by incubating 20 μl of the culture medium with 80 μl of lactate working solution at 37°C for 60 min, after which the absorbance at 450 nm was measured using the Bio-Rad iMark microplate reader. The absorbance at 450 nm was normalized to the respective protein concentration of sample (as measured by the BCA Assay kit; Thermo Fisher Scientific; 79023227).

### Statistical analysis

The sample size was not predetermined by statistical methods, but it is consistent with our previous publications. Statistical analyses were performed using GraphPad Prism version 10.4.0 software. First, normality distribution was assessed using the Shapiro-Wilk test. Statistical significance was determined using unpaired two-tailed Student’s *t* tests or Mann-Whitney test for two-group comparisons, and one-way or two-way analysis of variance (ANOVA) with post hoc tests for multiple-group comparisons. All data points represent biological replicates; samples were not measured repeatedly to obtain data. Kaplan-Meier survival analysis was conducted for time-to-death studies, with group differences assessed by the log-rank (Mantel-Cox) test. In animal experiments, each data point represents a single mouse, and experiments were randomized, though blinding was not implemented. Mice that became moribund before the end of the experimental time period were euthanized immediately, except for in survival studies. Results are presented as means with error bars indicating the SEM. *P* values less than 0.05 were considered statistically significant.
