# Supplementary material for: BCG vaccination mitigates tau pathology and restores cognitive function in PS19 mice
Source: Sci Adv. 2026 Oct 21;12(43):eaej4603. doi: 10.1126/sciadv.aej4603 (PMC13686014; doi:10.1126/sciadv.aej4603)
Supplement: Supplementary file 1 [file sciadv.aej4603_sm.pdf]

Science Advances
AAAS

Supplementary Materials for

BCG vaccination mitigates tau pathology and restores cognitive function in PS19 mice

Somnath Shee *et al.*

Corresponding author: William Bishai, wbishai@jhmi.edu

Sci. Adv. **12**, eaej4603 (2026)
DOI: 10.1126/sciadv.aej4603

The PDF file includes:

Table S1
Figs. S1 to S4
Legends for data S1 to S3

Other Supplementary Material for this manuscript includes the following:

Data S1 to S3

Institution	Subjects	% with BCG Tx	Hazard Ratio (% ↓ Alz. dementia)	p <0.05	F/U (yr)	Comment
**1. Six retrospective human studies of BCG for dementia**						
**Hebrew Unversity (Israel)**	1371	64%	0.282 (78%)	YES	14	
**MGH-Brigham (USA)**	6467	52%	0.8 (20%) Overall<br>0.7 (30%) Pt ≥ 70 yr	YES	15	-Decreased risk of death in patients without an earlier diagnosis of AD
**NYU, Montefiore (USA)**	1290	25%	0.41 (59%)	YES	3	-BCG effect was dose-dep.<br>-Females less responsive
**Hebrew Unversity (Israel)**	12,185	19%	0.72 (28%) HMO<br>0.42 (58%) Hospital	YES	3.5–7	-Parkinson's disease showed a reduction of 28% with BCG
**U. Washington Seattle (USA)**	26,584	51%	0.73 (27%)	YES	3–4	-NIH SEER database.<br>-BCG effect was dose-dep.
**Uppsala Univ., (Sweden)**	38,934	17%	0.88 (12%)<br>0.75 (25%) if age > 75	YES	6	-Swedish national database<br>-Females more responsive
**2. Small-scale prospective study**						
**Wisconsin, USA**	49 BCG-naïve healthy humans	100%		YES (0.016)	9 months	APS showed significant reduction in high-risk subjects (n = 10) after BCG doses.

Supplementary Table S1. Table explaining retrospective data, prospective data, meta-analysis demonstrating potential BCG-mediated beneficial effects against onset of dementia. (Tx- treated; F/U- Follow-up in years; APS- Amyloid probability score)

Step 1. Download all images from Proscia, with identical image parameters (Gamma correction, Brightness, Contrast, and Magnification).

Step 2. Load image in ImageJ.

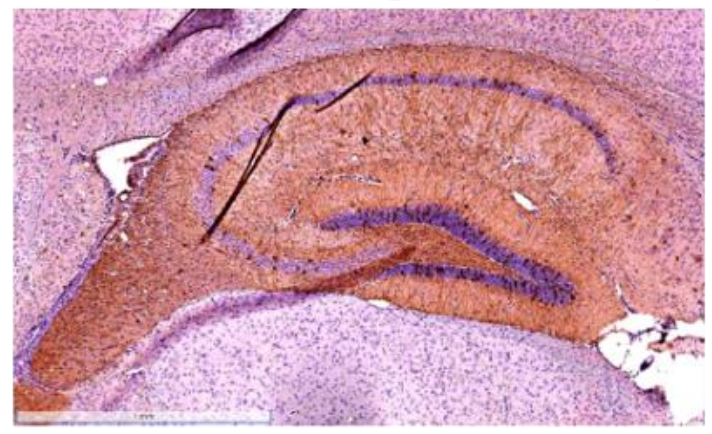

Step 3. Draw a line over the scale bar>Analyze>Set scale>Known distance= 1; Unit of length= mm; check “Global”> OK.

Step 4. Crop out scale bar.

Step 5. Image>color> colour deconvolution 2> Vectors: H&E DAB> OK

Step 6.

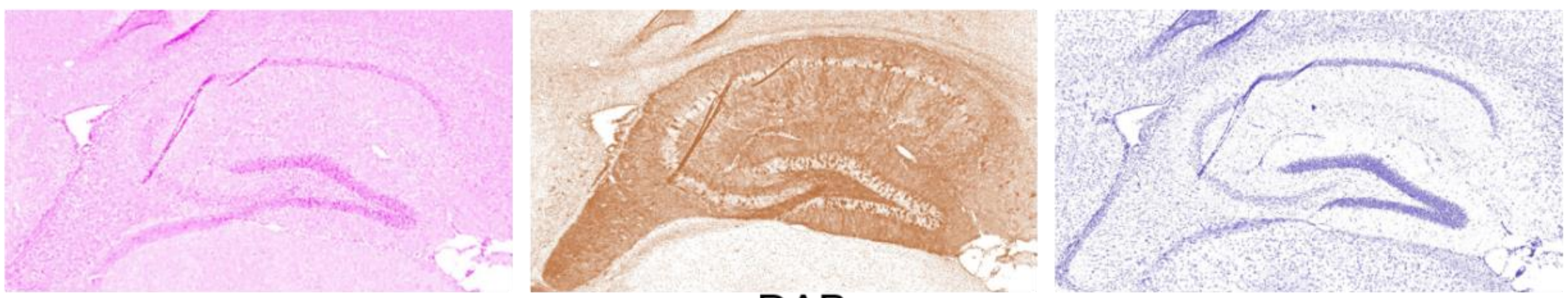

DAB

Step 7. Select DAB- image**.** Edit>invert.

Step 8. Select hippocampus area with polygon selection> Edit> Clear outside.

(Optional: Remove any obvious artifacts in the section> Select ROI with polygon selection> Edit> Clear.)

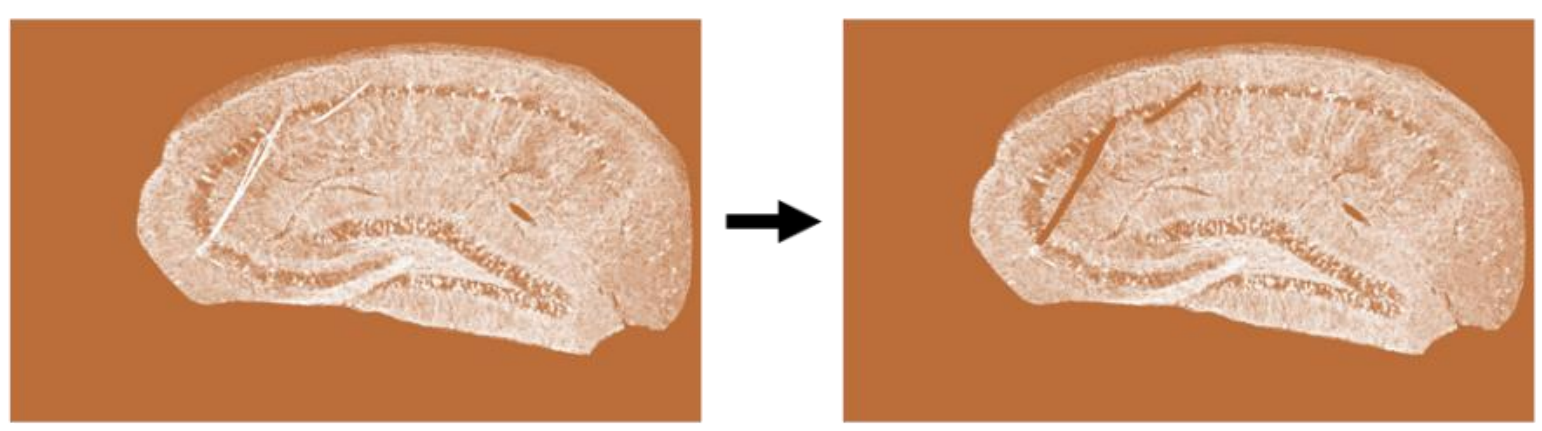

Step 9. Light/White shaded area should be AT8+ area. Using polygon selection, mark total hippocampus area.

Step 10. To remove false positives in the image, we selected strict threshold.

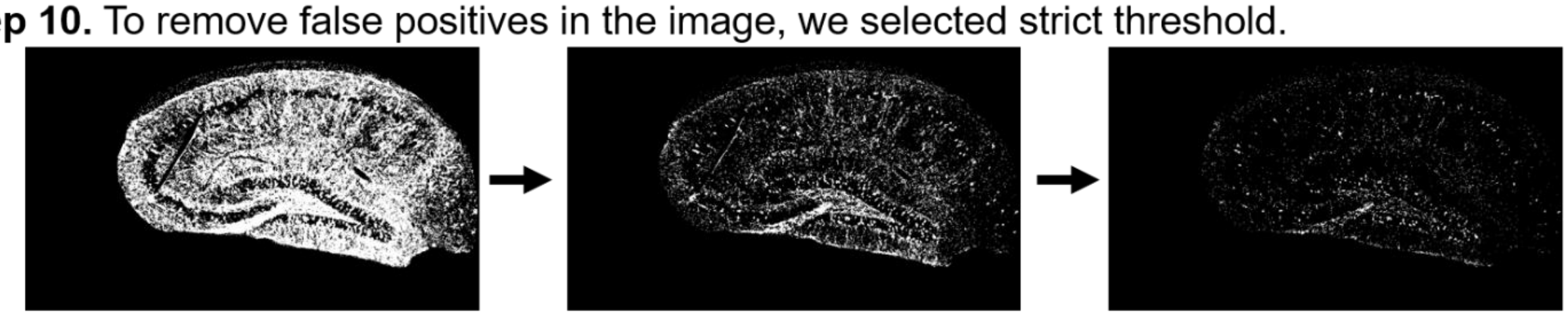

(Threshold= 133, 255)	(Threshold=200, 255)	(Threshold=225, 255)
%AT8+ area= 53.65	%AT8+ area= 13.02	%AT8+ area= 3.27

Step 11. Analyze>Measure> Record “%Area”.

Supplementary Figure S1. ImageJ-analysis pipeline used to quantify immunohistochemistry-positive area in the brain sections, as shown in Fig. 1. For each mouse brain sample, IHC was done in three planes (10 μm thick sections) for all the antibodies.

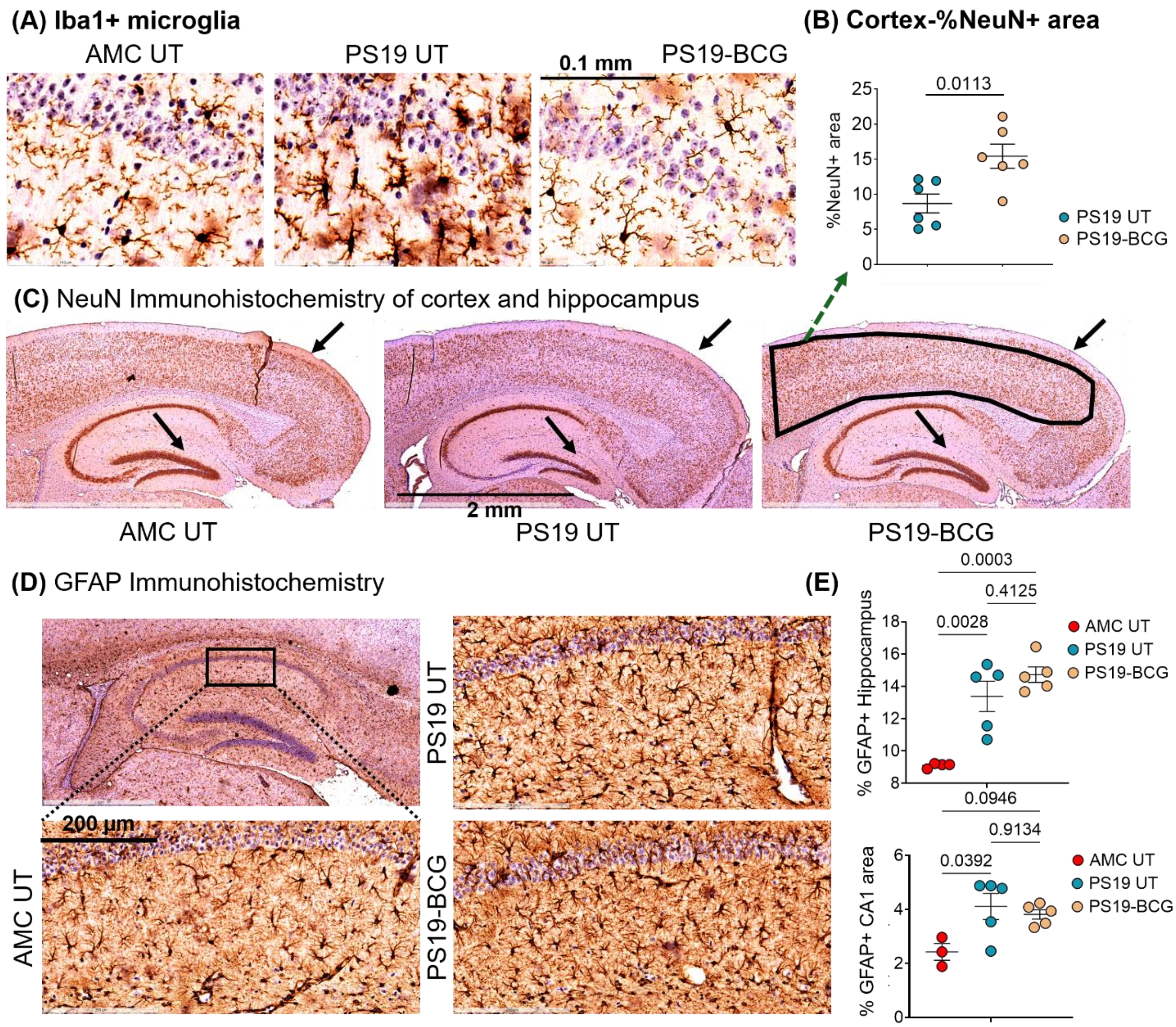

Supplementary Figure S2. ***M. bovis*** **BCG treatment ameliorates neurodegeneration, and microgliosis in PS19 mice, but did not reduce astrocyte activation levels. (A)** Representative image of Iba1-IHC area in the hippocampus-CA1 region of AMC UT, Sham (PBS) or BCG treated PS19 mice, showing restoration of homeostatic, ramified microglia in the BCG-treated PS19 mice. Fig. 1C is reused here and shown enlarged to highlight. **(B, C)** %NeuN+ area in the cortex region (somatosensory and visual area) of AMC UT, Sham (PBS) or BCG treated PS19 mice, as determined by ImageJ analysis. (n=6/group) Black arrow indicates differences in NeuN-IHC staining. **(D, E)** %GFAP+ area in the hippocampus and hippocampus-CA1 region of AMC UT, Sham (PBS) or BCG treated PS19 mice, as determined by ImageJ analysis. (n=6/group). The data are means ± SEM, and representative of two independent experiments. Each data point represents a mouse. Statistical analysis was calculated by unpaired two-tailed Student's t-test (B) and one-way ANOVA with Tukey's multiple comparisons test (E). (*p*>0.05: ns, *p*<0.01: **, *p*<0.001: ***, *p*<0.0001: ****)

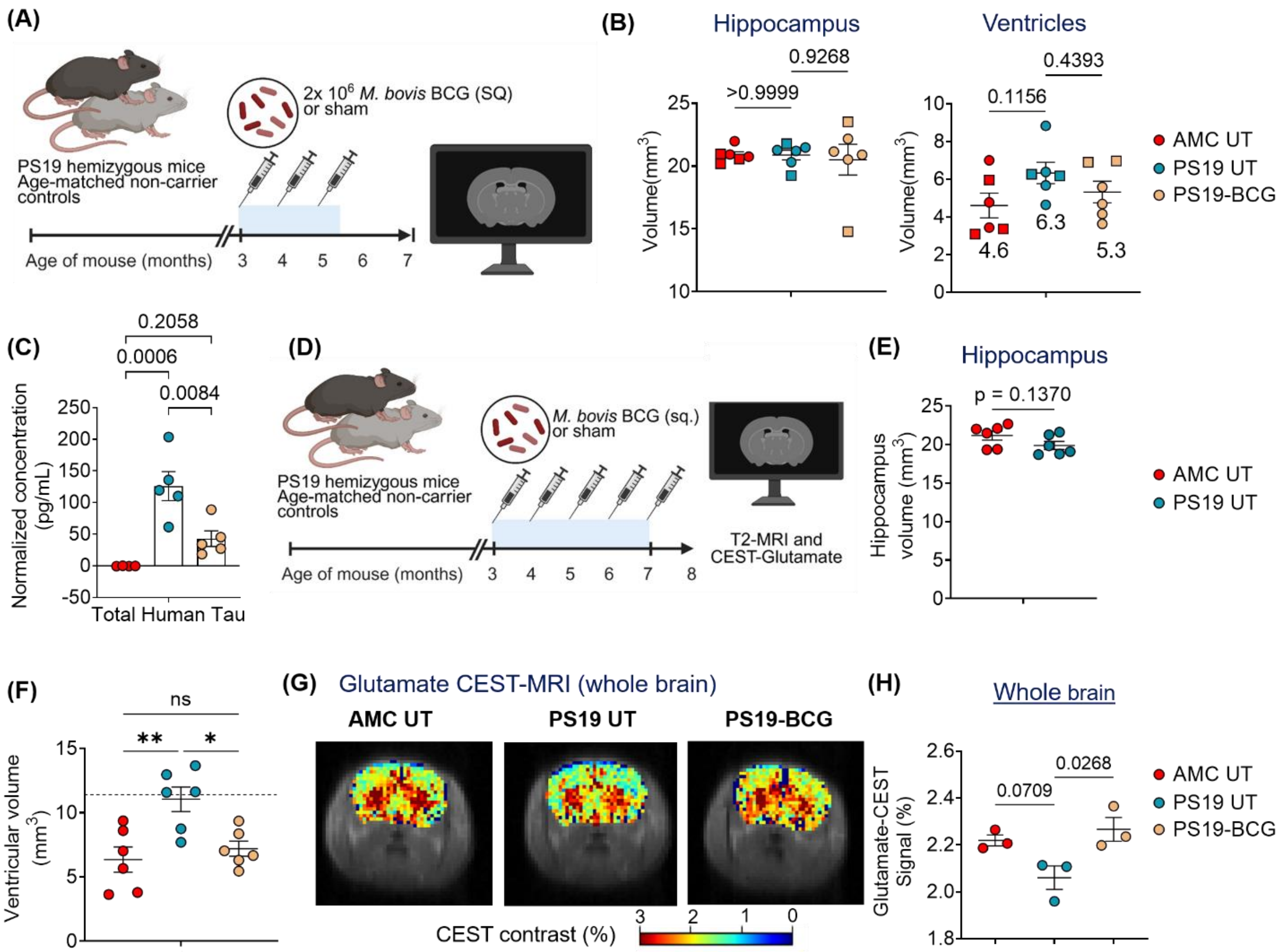

Supplementary Figure S3. *M. bovis* BCG treatment prevented ventricular dilatation in the brain of PS19 males in the cohort 2. (A) Schematic of the experiment-Cohort 1. **(B)** Quantitative analysis of the hippocampus and lateral ventricle volume. (n=6/group; 2-3 males (squares), 3-4 females(circles)) The data are means ± SEM. Each data point represents a mouse. Statistical analysis was calculated by one-way ANOVA with Sidak's multiple comparisons test. Mean ventricular volume are mentioned on the figure. **(C)** Total human tau protein levels detected in plasma from 6-7 months old PS19 mice. n= 5 mice/group. **(D)** Schematic of the experiment-Cohort 2. **(E, F)** Quantitative analysis of the hippocampus and lateral ventricle volume of PS19 males. n=6/group. **(G, H)** Representative image of glutamate-weighted chemical exchange saturation transfer (CEST-MRI) maps of the brain. (Cohort 1; n=3 mice/group), and quantification. The data are means ± SEM, and representative of two independent experiments. Each data point represents a mouse. Statistical analysis was calculated by unpaired two-tailed Student's t test (Hippocampus in **E**) and one-way ANOVA with Sidak's multiple comparisons test (**C, F, H**). (*p*>0.05: ns, *p*<0.01: **, *p*<0.001: ***, *p*<0.0001: ****) Schematics were created in BioRender. Shee, S. (2026) https://BioRender.com/kanmk73.

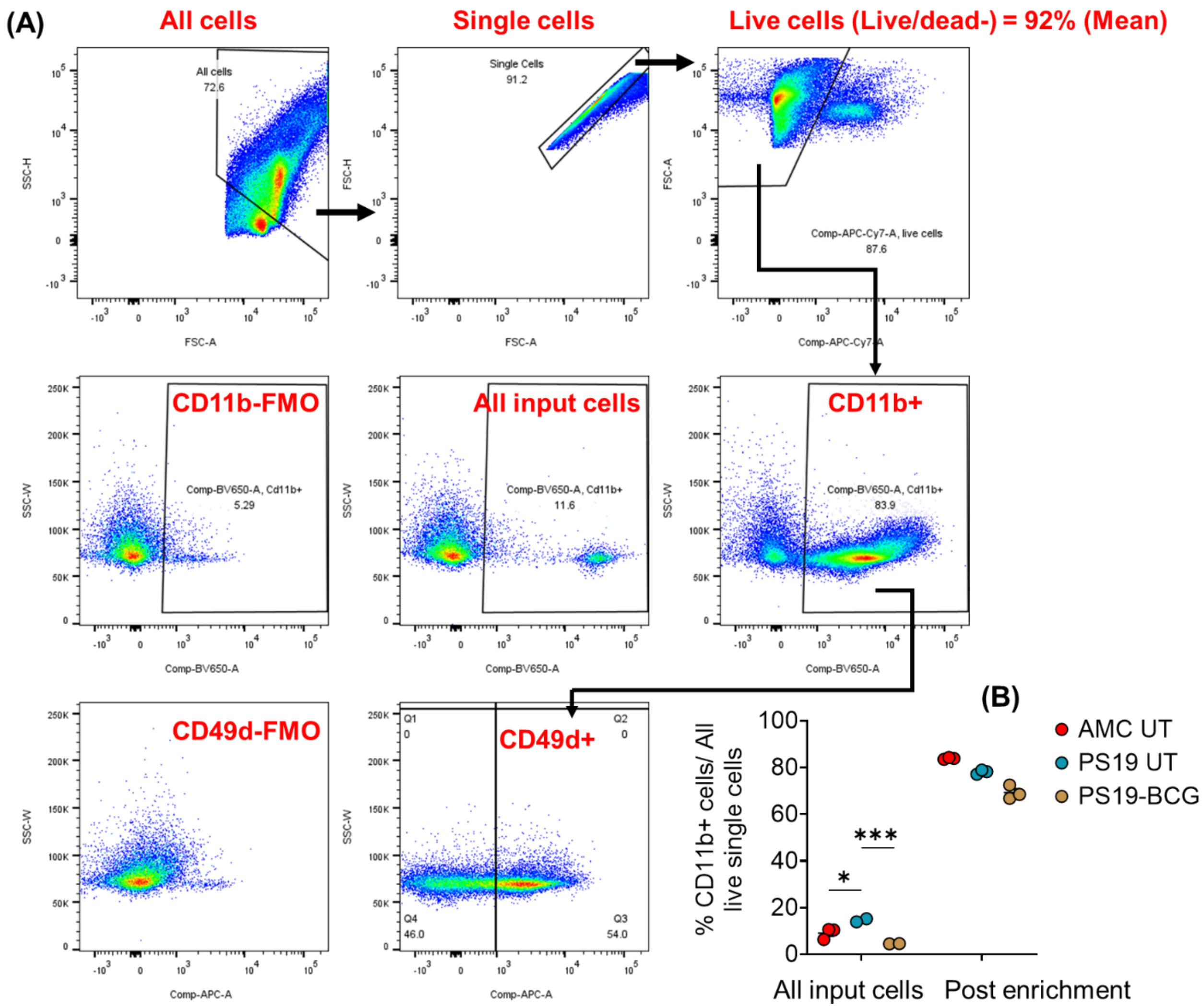

Supplementary Figure S4. Immunophenotyping of CD11b+ cells from PS19 mouse brain. (A) Representative flow cytometry dot plots and arrows depicting gating strategy and pre-gated cell populations. Gating for all the positively-stained events were established based on respective Fluorescence minus one (FMO) control. The mean viability of the CD11b+ cells derived from magnetic-bead based enrichment was 92%. **(B)** Higher CD11b+ cells out of all live single cells were detected in sham-treated PS19 brains, before the enrichment by magnetic beads (input cells). (n=6 mice/group; samples from 2 mice were pooled together) The data are means ± SEM, and representative of two independent experiments. Statistical analysis was calculated by two-way ANOVA with Tukey's multiple comparisons test. (*p*>0.05: ns, *p*<0.01: **, *p*<0.001: ***)

Table of contents for supplementary materials.

1. Data S1. Bulk RNA-Sequencing data of the PS19 hippocampus.
2. Data S2. Oligonucleotides used in the study.
3. Data S3. Source data file. Raw data used to generate graphs in the paper.
